# A Randomized Controlled Clinical Trial in Healthy Older Adults to Determine Efficacy of Glycine and N-Acetylcysteine Supplementation on Glutathione Redox Status and Oxidative Damage

**DOI:** 10.3389/fragi.2022.852569

**Published:** 2022-03-07

**Authors:** Giulia Lizzo, Eugenia Migliavacca, Daniela Lamers, Adrien Frézal, John Corthesy, Gerard Vinyes-Parès, Nabil Bosco, Leonidas G. Karagounis, Ulrike Hövelmann, Tim Heise, Maximilian von Eynatten, Philipp Gut

**Affiliations:** ^1^ Nestlé Institute of Health Sciences, Lausanne, Switzerland; ^2^ Profil, Neuss, Germany; ^3^ Nestlé Institute of Food Safety and Analytical Sciences, Lausanne, Switzerland; ^4^ Nestlé Health Science, Vevey, Switzerland; ^5^ Institute of Social and Preventive Medicine, University of Bern, Bern, Switzerland

**Keywords:** glycine, n-acetylcysteine, glutathione, total cysteine, cardiometabolic diseases, healthy aging, oxidative stress, nutrition

## Abstract

Glycine and cysteine are non-essential amino acids that are required to generate glutathione, an intracellular tripeptide that neutralizes reactive oxygen species and prevents tissue damage. During aging glutathione demand is thought to increase, but whether additional dietary intake of glycine and cysteine contributes towards the generation of glutathione in healthy older adults is not well understood. We investigated supplementation with glycine and n-acetylcysteine (GlyNAC) at three different daily doses for 2 weeks (low dose: 2.4 g, medium dose: 4.8 g, or high dose: 7.2 g/day, 1:1 ratio) in a randomized, controlled clinical trial in 114 healthy volunteers. Despite representing a cohort of healthy older adults (age mean = 65 years), we found significantly higher baseline levels of markers of oxidative stress, including that of malondialdehyde (MDA, 0.158 *vs.* 0.136 µmol/L, *p* < 0.0001), total cysteine (Cysteine-T, 314.8 *vs.* 276 µM, *p* < 0.0001), oxidized glutathione (GSSG, 174.5 *vs.* 132.3 µmol/L, *p* < 0.0001), and a lower ratio of reduced to oxidized glutathione (GSH-F:GSSG) (11.78 *vs.* 15.26, *p* = 0.0018) compared to a young reference group (age mean = 31.7 years, *n* = 20). GlyNAC supplementation was safe and well tolerated by the subjects, but did not increase levels of GSH-F:GSSG (end of study, placebo = 12.49 *vs.* 7.2 g = 12.65, *p*-value = 0.739) or that of total glutathione (GSH-T) (end of study, placebo = 903.5 *vs.* 7.2 g = 959.6 mg/L, *p*-value = 0.278), the primary endpoint of the study. Post-hoc analyses revealed that a subset of subjects characterized by high oxidative stress (above the median for MDA) and low baseline GSH-T status (below the median), who received the medium and high doses of GlyNAC, presented increased glutathione generation (end of study, placebo = 819.7 *vs.* 4.8g/7.2 g = 905.4 mg/L, *p*-value = 0.016). In summary GlyNAC supplementation is safe, well tolerated, and may increase glutathione levels in older adults with high glutathione demand.

Clinical Trial Registration: https://clinicaltrials.gov/ct2/show/NCT05041179, NCT05041179.

## Introduction

Reactive oxygen species (ROS) are a group of short-lived oxygen-containing molecules formed mainly as byproducts of oxidative metabolism in mitochondria (Beckman and Ames, 1998). Due to their reactive nature, oxidative stress from ROS can damage macromolecules such as DNA and structural proteins, organelles, and interfere with biological processes including mitochondrial energy generation, cellular signaling, autophagy, senescence, and apoptosis (Beckman and Ames, 1998; Lee et al., 2012; Shadel and Horvath, 2015; Sies, 2015; Garcia-Prat et al., 2016). Intracellular ROS concentrations can reach damaging levels when they are not adequately detoxified by cellular antioxidants, a mechanism that is thought to contribute to the cellular aging process and the onset of chronic diseases (Harman, 1992; Sies, 1997; Beckman and Ames, 1998; Forrester et al., 2018; Redman et al., 2018). Several cellular enzymes, including superoxide dismutases, catalases, and glutathione peroxidases (GPXs) as well as low molecular antioxidants from dietary intake or biosynthesis are the principal mechanisms to maintain ROS levels within a physiological range (Sies, 1997).

The tripeptide glutathione (GSH) is the main low molecular antioxidant ubiquitously present in cells where it is produced from the amino acids cysteine, glycine, and glutamate (Sies, 1997; Sies, 1999; Wu et al., 2004; Forman et al., 2009). Monomeric GSH takes part in a reduction-oxidation (redox) buffer system together with its oxidized form glutathione disulfide (GSSG). In this reaction, reactive oxygen molecules are neutralized by a reducing equivalent from the thiol group of GSH, which in turn is oxidized to form the dimer GSSG through a process that is regulated by GPXs (Sies, 1999; Wu et al., 2004; Forman et al., 2009). Glutathione concentrations are in the low micromolar range in plasma but reach levels between 0.5 and 10 mM in cells, including erythrocytes and other circulating blood cells (Wu et al., 2004; Forman et al., 2009). The ratio of reduced GSH to oxidized GSSG is enzymatically maintained at >10 by glutathione reductase and NADPH in physiological conditions and is dependent on the tissue type (Sies, 1999; Wu et al., 2004). In addition, glutathione synthesis rates across tissues vary, likely reflecting different demands in steady state and in response to stressors, such as acute inflammation (Malmezat et al., 2000). A chronically diminished GSH:GSSG ratio in circulating blood is however thought to be a valuable surrogate marker of elevated oxidative stress in conjunction with the appearance of low-molecular oxidation products, such as lipid peroxides and protein or non-protein bound forms of cysteine disulfides in blood plasma (Sies, 1999; Wu et al., 2004; Ho et al., 2013; Fu et al., 2019).

GSH levels have been reported to decline with old age (Lang et al., 1992; Samiec et al., 1998; Lang et al., 2000; Sekhar et al., 2011) and to be lower in a number of chronic medical conditions associated with oxidative stress, including diabetes (Martina et al., 2008), hypertension (Hildebrandt et al., 2015), macular degeneration (Samiec et al., 1998), cystic fibrosis (Roum et al., 1993), chronic obstructive pulmonary disease (COPD) (Zheng et al., 2014), HIV (Herzenberg et al., 1997), COVID-19 (Kumar et al., 2021b), and inherited mitochondrial diseases (Hargreaves et al., 2005), among others (Rushworth and Megson, 2014). In addition, genetic variants in genes encoding for GPXs have been associated with increased mortality from cardiovascular diseases (Zhang et al., 2014) and diabetes (Hamanishi et al., 2004). GSH further has a role in the detoxification of acetaminophen poisoning by forming conjugates with the toxic intermediate n-acetyl-p-benzoquinone (Lauterburg et al., 1983). Intravenous administration of N-acetylcysteine (NAC), an acetylated form of cysteine with improved pharmacokinetic properties (Borgstrom and Kagedal, 1990), restores glutathione levels in the liver and is used as an antidote for acetaminophen-induced acute liver failure (Lauterburg et al., 1983; Smilkstein et al., 1988). The concept of glutathione as an essential guardian for cellular protection from oxidative stress and xenobiotic toxicity has spurred a large number of clinical studies using NAC in different chronic disease conditions (Sies, 1999; Townsend et al., 2003). For example, NAC alone, or in combination with glycine, have been reported to restore glutathione status and improve disease outcomes in patients with HIV (Herzenberg et al., 1997; De Rosa et al., 2000; Nguyen et al., 2014; Gupta et al., 2016; Kumar et al., 2020) and COPD (Zheng et al., 2014; Hirai et al., 2017). In addition, clinical trials in small cohorts of elderly with insulin resistance and high pro-inflammatory markers showed promising effects using NAC and glycine supplementation on a lowering of oxidative stress markers as well as improved endpoints related to metabolic, physical, and cognitive health (Sekhar et al., 2011; Nguyen et al., 2013; Kumar et al., 2021a).

Despite this body of evidence that points to a role of impaired glutathione levels in age-related chronic diseases, much less is currently known about glutathione status and levels of oxidative stress in healthy subjects. Specifically, information on glutathione status in older adults considered generally healthy is currently limited and therefore the benefits of GlyNAC supplementation in such a population are not well established.

Here, we aimed to assess the efficacy and safety of GlyNAC in a randomized controlled clinical trial in healthy older adults (60–85 years) recruited to represent healthy aging in the absence of disabling chronic medical conditions. The primary outcome was the level of total glutathione (GSH-T) in whole blood compared to placebo at the end of the study. 117 participants from 60 to 85 years were enrolled at a single center, receiving placebo or GlyNAC at 2.4, 4.8, or 7.2 g (1:1 ratio) divided in two doses per day for 14 days. Dosing efficacy as well as circulating levels of GSH-T, the ratio of reduced to oxidized glutathione (GSH-F:GSSG) and concentrations of malondialdehyde (MDA), a lipid peroxide marker of oxidative stress, as well as that of total cysteine, an indicator of oxidation of reduced cysteine to oxidized forms of cysteine, were assessed as secondary efficacy outcomes. In addition, safety and tolerability of GlyNAC were monitored. A group of 20 young healthy volunteers (mean age = 31.7 years) was recruited for comparison of outcomes with the older participants.

## Materials and Methods

### Ethics and Registration

The trial was conducted in compliance with the International Conference on Harmonization (ICH) guidelines and the ethical principles of the Declaration of Helsinki and its subsequent amendments. The trial design was approved by the IEC/IRB of North Rhine Medical Council (IRB number: 2018188). All trial procedures were conducted by Profil Institute for Metabolic Research GmbH (Neuss, Germany) in collaboration with MLM Medical Labs GmbH (Moenchengladbach, Germany). The trial is registered at clinicaltrial.gov (NCT05041179).

### Inclusion and Exclusion Criteria

**FIGURE 1:**
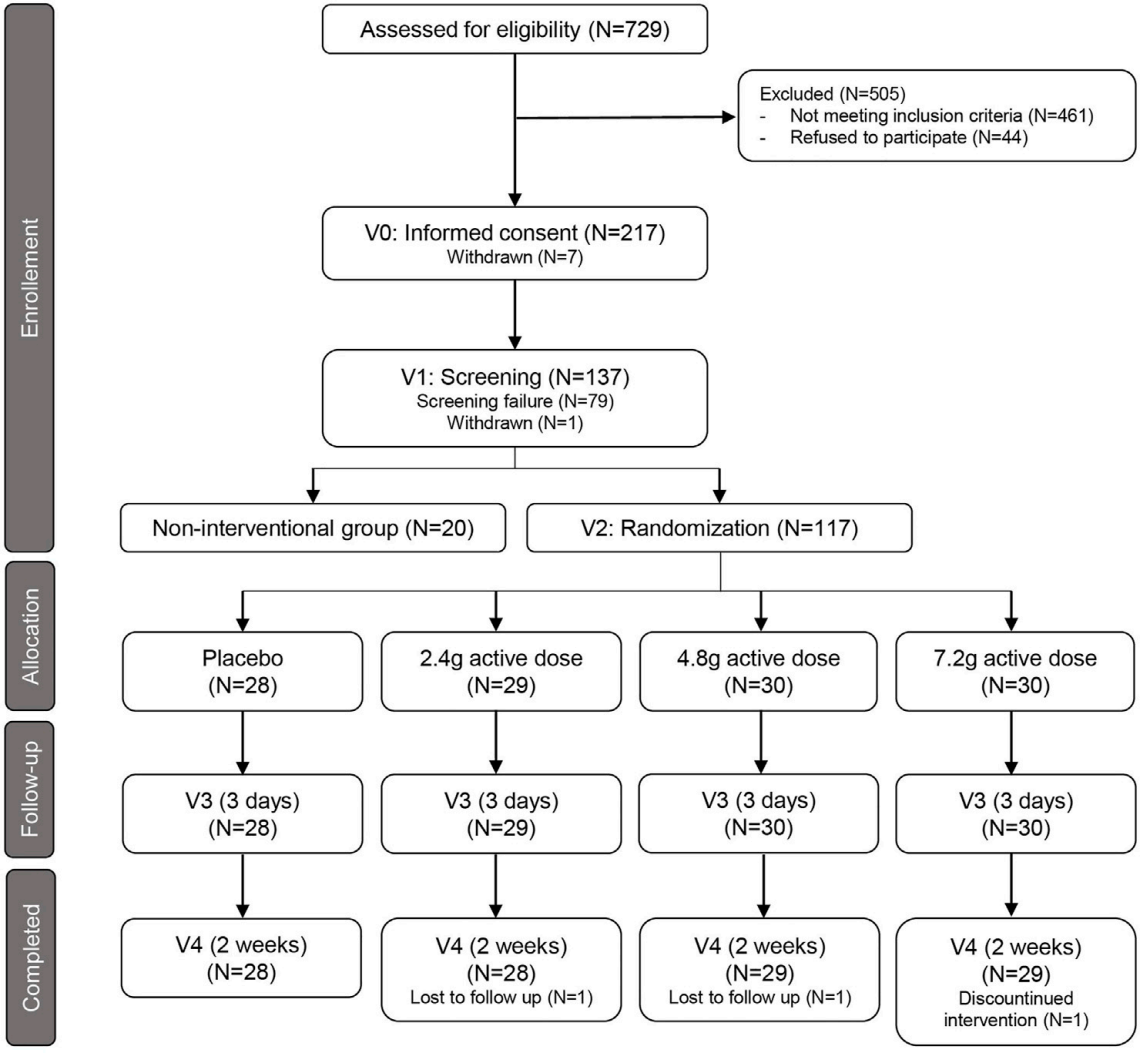
Flowchart of study design. Summary of recruitment process of healthy young subjects and healthy older adults and their randomization to the placebo arm and the three treatment groups. Follow-up indicates the interim time point at 3 days after first dosing (V3). V= Visit; N= Numbers.

The study flowchart is shown in Figure 1. 729 participants were voluntarily recruited from the database and a written informed consent was obtained from 217 subjects during an ambulatory visit (Visit 0), where eligibility and willingness to participate were assessed. Inclusion criteria for eligibility were: age range 20–40 years old (young adults) and 60–85 years old (older adults), both inclusive; both sexes were admitted; BMI range 18.5–30 kg/m^2^ (young adults) and 25–35 kg/m^2^ (older adults), both inclusive; considered generally healthy; sedentary for older adults, with less than 1 h of strenuous exercise per week; levels of HbA1c less than 5.7% (38.8 nmol/mol for young adults) and 6.5% (47.5 nmol/mol for older adults). Subjects with known clinically relevant comorbidity, acute illness, serious infectious disease for 4 weeks prior the product intake, abnormal screening laboratory tests, and abnormal blood pressure (systolic blood pressure <90 mmHg or >139 mmHg and/or diastolic blood pressure <50 mmHg or >89 mmHg) were excluded, although subjects with normal blood pressure taking anti-hypertensive medication were included. Heavy smokers (more than five cigarettes per day) and people with history of alcoholism or drug abuse were also excluded and participants were asked to refrain smoking 3 days prior and during the intervention. Participants were also asked to avoid any medication for 14 days before test product intake, consumption of high protein supplements within 60 days of screening and during the study, and consumption of any antioxidant, vitamins, herbal supplements within 2 weeks prior to and during the study. The full list of exclusion criteria is available in Supplementary Table S2. Conformity of participants to all exclusion criteria was assessed during the screening visit (Visit 1), during which a clinical examination and a blood sample was taken; a total of 137 participants were selected to enter the study (20 young adults, non-interventional group; 117 older adults, interventional group).

### Study Protocol

The study is a single-center, double-blind, randomized, placebo-controlled 4-arm study design. Following the screening visit, the interventional group of older adults have undergone three visits on day 1 (Visit 2), day 4 (Visit 3), and day 15 (Visit 4) of the treatment supplementation. At Visit 2, participants were randomized in a 1:1:1:1 ratio in four different arms: placebo, 2.4 g of actives, 4.8 g of actives, 7.2 g of actives. Participants came to each visit after an overnight fast (>10 h), with up to 200 ml of water allowed, avoiding strenuous exercise within the last 48 h, avoiding a protein-rich meal in the evening before the visit as well as consumption of alcohol, caffeine and/or methylxanthine-containing products in the last 24 h before the visits. At each visit the subjects had a medical examination (body measurements, vital signs, and physical examination). Two blood samples were taken 60 min before and 60 min after the product intake for assessment of glycine, total cysteine, GSH-T, GSH-F, GSH-F:GSSG in whole blood and glycine, total cysteine, cystine, MDA in plasma. Subjects received a mixed meal (breakfast) after the product intake.

### Product Dosing, Composition, and Administration

A powder blend of N-acetylcysteine (NAC) and glycine was tested at three different daily doses: 2.4 g of actives (1.2 g NAC + 1.2 g glycine), 4.8 g of actives (2.4 g NAC + 2.4 g glycine), and 7.2 g of actives (3.6 g NAC + 3.6 g glycine) per day. Each dosage was split into two doses consumed in the morning and in the evening. The placebo control arm received 7.2 g of isomaltulose with the same way of administration. In order to keep it blind, all subjects took three sachets of test product at each dosing; all sachets had the same weight of 1.2 g. The scheme of the sachet composition for each arm is shown in Supplementary Table S1. The investigational product was manufactured, filled, and labelled by Glanbia Nutritionals GMBH, Germany. Secondary and tertiary packing of the study product for blinding purposes was performed at Profil Institute.

### Glutathione, Cysteine, Glycine, and MDA Measurements

GSH-T, GSH-F, GSH-F:GSSG, total cysteine and glycine levels were measured in whole blood. After blood withdrawal into EDTA, samples were rapidly stored at −80°C. The quantitative analysis of free GSH and GSH-T was performed according the applicable international standards from the FDA and EMA, using the HPLC method from Chromsystems (Gräfelfing/München, Germany). Protein precipitation was performed on all samples, standards and controls. Prior to analysis, samples underwent a protein precipitation step followed by a derivatization step. To detect both forms of glutathione (GSH-F and GSSG), each sample is divided into two aliquots after the initial protein precipitation. For detection of GSH-F, the first aliquot is derivatized immediately. The second aliquot is first chemically reduced before derivatization, which leads to the conversion of GSSG to GSH. In this second aliquot the sum of both oxidized and reduced glutathione (GSH-T) can be analyzed. The subsequent analysis bases on HPLC determination with fluorescence detection. The concentration of the oxidized form GSSG was calculated from the obtained results using the formula GSSG = GSH-T - GSH-F x 0.99672. A similar method was used for the MDA quantification, assessed also by HPLC Chromsystems (Gräfelfing/München, Germany). The analysis was performed using the Chromsystem reagent kit, that allows the determination of MDA on an isocratic HPLC system with fluorescence detection. Before the fluorescence detection, EDTA plasma samples and controls were prepared with a precipitation step followed by derivatization.

Quantification of glycine, total cysteine, cystine and NAC in plasma was performed with Ultra Performance Liquid Chromatography tandem Mass Spectrometry analysis (UPLC-MS/MS). Samples, standards and controls were added with internal standards working solution and tris (2-carboxyehtyl) phosphine hydrochloride solution (Merck KGaA, Darmstadt, Germany), or replace by Milli-Q water for cystine measurements, and vortexed at 800rpm for 15 min at room temperature. Protein precipitation using methanol (Merck KGaA, Darmstadt, Germany) and 0.1% formic acid (Merck KGaA, Darmstadt, Germany) was performed and the collected supernatant was treated with a derivatization solution of aminoquinolyl-N-hydroxysuccinimidyl carbamate (AQC) (Merck KGaA, Darmstadt, Germany). After incubation at 55°C, a final dilution of the derivatized mixture was done by an addition of ammonium formate solution 0.55 g/L (Merck KGaA, Darmstadt, Germany) in water containing 0.1% formic acid (Merck KGaA, Darmstadt, Germany). The samples were then injected into the UPLC-MS/MS for analysis. Chromatographic separation for cystine measurements was performed using AccQ-TAG Ultra C18 100 × 2.1 1.7 um analytical column (Waters^®^, United States), for glycine, cysteine, and NAC using an UPLC YMC-Triart metal Free C18 50 × 2.1 1.9 um analytical column (YMC^®^, Japan). Mass spectrometric analysis and detection were performed using specific MRM for analytes and their related labeled internal standards, on a Xevo TQ-XS (Waters^®^, United States), equipped with electrospray ionization source in positive mode and argon as collision gas. Integration of chromatographic peaks was performed using Masslynx software (Waters^®^, United States). The analytical method for the quantification of glycine in whole blood is based on precipitation, centrifugation and dilution of the supernatant, followed by the determination of the glycine using Liquid Chromatography tandem Mass Spectrometry analysis (LC-MS/MS). Samples, standards and quality controls were added with internal standards working solution and precipitated with acetonitrile (Chemsolute, Th.Geyer, Renningen, Germany) and 0.1% formic acid (Merck KGaA, Darmstadt, Germany). The supernatant was transferred in a 96-well plate and submit to LC-MS/MS analysis using Mass Spectrometer TSQ Vantage (Thermo Fisher Scientific, Waltham, Massachusetts, United States). The time point of 60 min to determine oral bioavailability of the interventional product was decided based on the published t_max_ of approximately 45 min after oral ingestion of NAC (Borgstrom and Kagedal, 1990) and that of approximately 75 min of glycine in blood plasma of healthy volunteers (Butterworth et al., 1958).

### Safety Panels

Safety of the product was assessed during the study through the monitoring of adverse events and blood biochemistry analysis performed by MLM Medical Labs. Basal blood hematology and biochemistry were performed at the screening Visit 1 on all subjects. At the end of the study at Visit 4, a short list of hematology and biochemistry markers was performed to monitor the safety of the product intake at−60 min before and 120 min after the product intake. The parameters assessed were: hematocrit, hemoglobin, erythrocytes, mean corpuscular volume (MCV), mean corpuscular hemoglobin (MCH), MCH concentration, platelets, leucocytes, sodium, potassium creatinine, glucose, total cholesterol, aspartate aminotransferase (GOT), alkaline phosphatase, gamma-glytamyltransferase (ɣ-GT), insulin, triglycerides. An exhaustive list of all parameters detected can be find in Annex 1.

### Statistical Methods

The sample size calculations for the interventional cohort are based on unpublished data from Profil which evaluated total GSH measurement in healthy young subjects. A sample size of 120 subjects (4 arms; 30 per arm) would provide 80% power to detect a significant intraindividual change in total GSH of 12.5% (comparison baseline to end-of-treatment) and significant interindividual difference between groups (0 vs 2.4 *vs.* 4.8 *vs.* 7.2 g) of 17.5% at *α* = 0.05 level. To account for potential outliers, an additional eight subjects (2 per arm) results in a total sample size of N = 128. For the non-interventional cohort, a group size of 20 subjects will detect a minimum difference in baseline GSH of at least 16% between young and elderly healthy subjects (0.05 alpha, 0.8 power) based on an inter-subject CV of 22%. Demographic and general baseline characteristics are represented as LS-means with standard deviations for normally distributed parameters or medians with IQR for non-normally distributed parameters. The baseline values of the young adults were compared to the baseline parameters of older adults using parametric t-statistics for LS-means and one-tailed nonparametric Wilcoxon/Mann-Whitney tests for medians. Correlation analysis between baseline parameters were performed in older adults with all arms pooled. A linear mixed model was used to test intraindividual changes from baseline (Visit 2) and end of study (Visit 4) within each dose group and from placebo group. All hypothesis tests were assessed at 5% level.

### Data Availability Statement

Data related to this study as well as a clinical study report released by Profil and Nestlé Health Science are available upon reasonable request.

## Results

### Cohort Characteristics and Levels of Oxidative Stress Markers at Baseline

**FIGURE 2:**
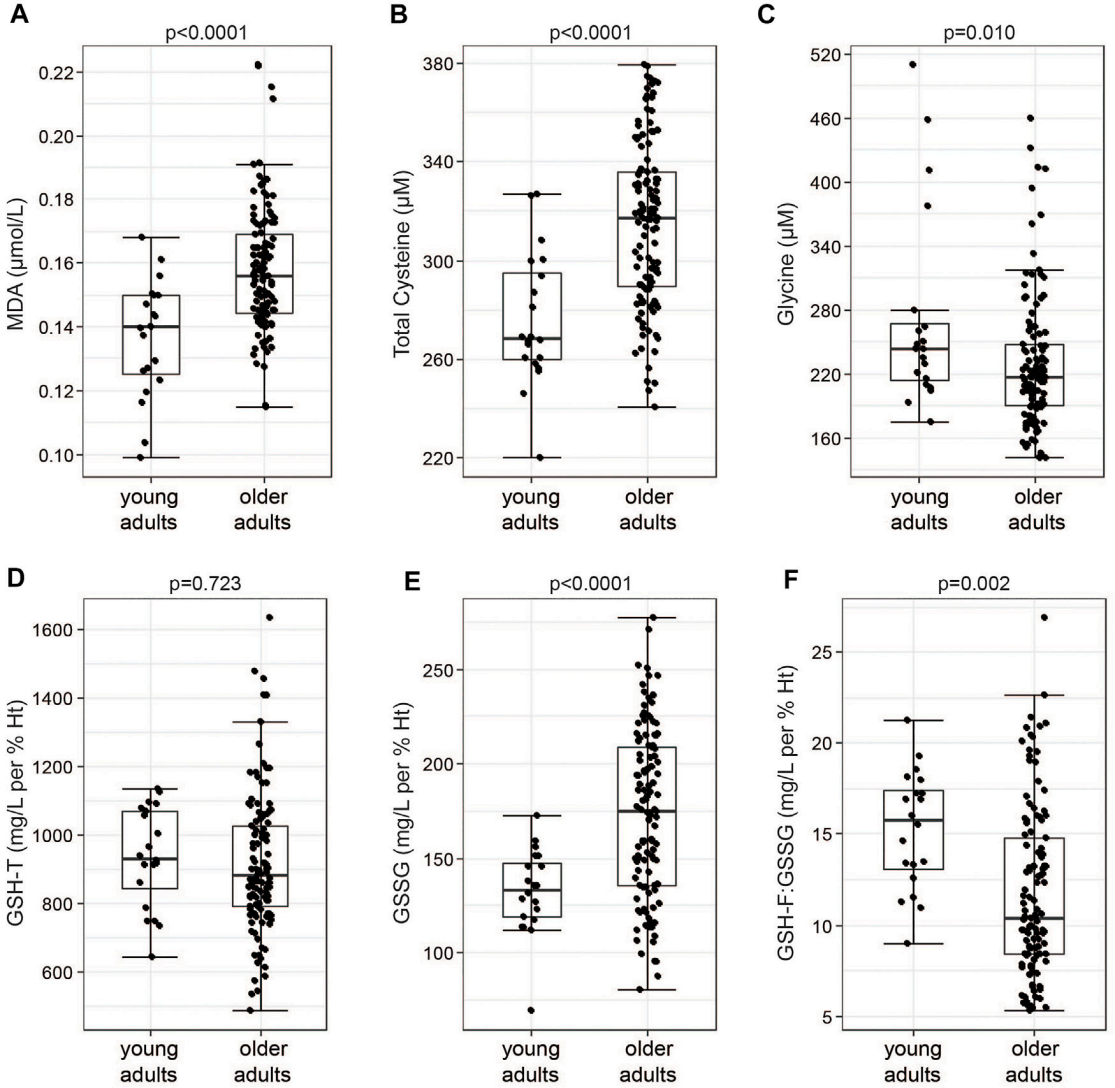
Healthy older adults show increased levels of markers of oxidative stress. **(A)** Levels of MDA, **(B)** total cysteine and **(C)** glycine measured in plasma samples of the study volunteers. **(D)** Levels of GSH-T, **(E)** GSSG and **(F)** free to oxidized glutathione (GSH-F:GSSG) in whole blood samples. Boxplot shows the median, the first to third quartile, the 1.5x interquartile ranges, and outliers. *p* = *p*-values, parametric t-statistics of LS-means.

117 older adults between 60 and 85 years of age were enrolled and randomized into a placebo group and three groups receiving active doses at 1.2, 2.4, and 3.6 g of each of Gly and NAC daily (Figure 1; Supplementary Table S1). 114 of the participants completed 2 weeks of daily GlyNAC administration orally taken in two doses (Figure 1). The cohort consisted of generally healthy older adults: subjects were excluded if they had hypertension (>139/89 mmHg), if they had a fasting glycated hemoglobin A1c (HbA_1c_) > 6.5% (47.5 nmol/mol), indicative of diabetes, a diagnosis of diabetes, a BMI >35 kg/m^2^, or if they suffered from other major chronic medical conditions, such as dementia, frailty, or malignancies (Supplementary Table S2). A non-interventional control cohort of 20 healthy adults between 20 and 40 years of age was recruited as a reference group. Baseline characteristics of the non-interventional cohort of young adults and the interventional cohort of older adult volunteers are summarized in Table 1. Several markers related to metabolic health, including HbA_1c_, triglycerides, LDL, total cholesterol, and C-reactive protein (CRP) were moderately increased in healthy older adults. The mean BMI of 28 kg/m^2^ is in the range of overweight category but is within the normal distribution in a healthy cohort of older adults (Peng et al., 2019). Consistent with a moderate increase of these well-described changes in healthy aging, we observed significantly higher levels of malondialdehyde (MDA), a marker for circulating levels of lipid peroxides, in older adults compared to the levels of young subjects (means, young = 0.136, older adults = 0.158 µmol/L, *p*-value < 0.0001) (Table 1; Figure 2A). We also observed higher levels of total cysteine in plasma (means, young = 276, older adults = 314.8 µM, *p*-value < 0.0001) (Figure 2B), representing the pool of reduced cysteine, cysteine disulfides and protein-bound cysteine after addition of the reducing agent dithiothreitol (DTT) (Fu et al., 2019). Together with these changes, we identified significantly lower levels of circulating glycine in blood plasma (means, young = 271.6, older adults = 229.4 µM, *p*-value = 0.01) (Figure 2C). We next analyzed the status of the glutathione redox system in whole blood. The levels of GSH-T representing the combined pool of reduced and oxidized glutathione as well as protein-bound glutathione were unaffected in older adults compared to that of young subjects (Figure 2D). However, despite normal levels of GSH-T, the oxidized glutathione (GSSG) levels were significantly increased leading to a lower GSH-F:GSSG ratio in older compared to young adults (Table 1; Figures 2E,F). While GSH-F:GSSG and GSH-T levels were tightly correlated, the oxidative damage markers assessed in this study did not correlate with glutathione status (Supplementary Figure S1). These data show that older age in an overall healthy cohort is associated with higher levels of oxidative stress markers indicating a shift towards a pro-oxidative redox balance.

**TABLE 1:** Baseline characteristics of study participants. Anthropometric and metabolic characteristics of young (non-interventional cohort) and older (interventional cohort) study subjects and differences in oxidative stress-related markers.

	Young adults	Older adults	*P (young vs older)*
	*Count (F/M)*	*Count (F/M)*
*n*	20 (9/11)	117 (64/53)	-
	*Mean (sd)*	*Mean (sd)*	
Age (yr)	31.7 (5.71)	65.5 (4.49)	-
Body weight (kg)	71.26 (11.83)	83.5 (10.45)	< 0.0001
BMI (kg/m^2^)	23.81 (3.06)	28.89 (2.79)	< 0.0001
HbA_1c_ (%)	5.20 (0.25)	5.66 (0.28)	< 0.0001
Fasting plasma glucose (mmol/l)	n.d.	5.61 (0.49)	-
Fasting plasma insulin (pmol/l)	n.d.	9.27 (5.72)	-
HOMA-IR	n.d.	2.34 (0.14)	-
ISI (composite)	n.d.	118.2 (6.16)	-
Triglycerides (mmol/L)	0.868 (0.406)	1.284 (0.544)	0.002
HDL cholesterol (mmol/L)	1.375 (0.275)	1.508 (0.365)	0.151
LDL cholesterol (mmol/L)	3.116 (0.814)	3.759 (0.920)	0.002
Glycine normalized to hematocrit	887.6 (257.56)	808.3 (219.67)	0.148
Cysteine-T normalized to hematocrit	319.8 (48.41)	415.4 (76)	<0.0001
Glycine in plasma (µM)	271.6 (92.3)	229.4 (61.67)	0.01
Cysteine-T in plasma (µM)	276 (26.86)	314.8 (33.54)	<0.0001
GSH-T normalized to hematocrit (mg/L)	938.1 (146.51)	921.5 (205.34)	0.73
GSH-F: GSSG normalized to hematocrit	15.26 (3.24)	11.78 (4.69)	0.002
MDA (µmol/L)	0.136 (0.018)	0.158 (0.019)	<0.0001
	*Median [IQR]*	*Median [IQR]*	
C-reactive protein (mg/L)	0.4 [0.725]	1.6 [1.9]	<0.0001

Means are compared using parametric t statistics and median using nonparametric Wilcoxon/Mann–Whitney tests. Cysteine-T, total cysteine disulfides; GSH-T, total glutathione; GSH-F, free reduced glutathione; ISI, insulin sensitivity index; MDA, malondialdehyde.

### Safety

No severe adverse effects were reported during the course of the study and none of the study subjects discontinued GlyNAC because of adverse effects. Two of the three subjects that discontinued the study opted to not participate in Visit 4 because of COVID-19 recommendations to reduce unnecessary travels. One subject discontinued because of high blood pressure. A comprehensive safety panel of relevant blood markers was quantified from each study subject at the beginning of the study, at 3 days after first dosing and after 2 weeks of GlyNAC consumption, including blood pressure, glucose, insulin, creatinine, Alkaline Phosphatase (ALP), Alanine Aminotransferase (ALT), among others (Supplementary Table S3; Supplementary Data). No significant effects could be observed for any of the markers except for clinically not relevant, but significant changes in ALP (placebo = 60.67; 2.4 g = 65.26, *p*-value = 0.216; 3.6 g = 70.66, *p*-value = 0.009; 7.2 g = 70.52, *p*-value = 0.01, U/L, compared to placebo). In conclusion, 2 weeks of daily GlyNAC consumption is safe and well tolerated.

### Dose-Dependency of GlyNAC Bioavailability

**FIGURE 3:**
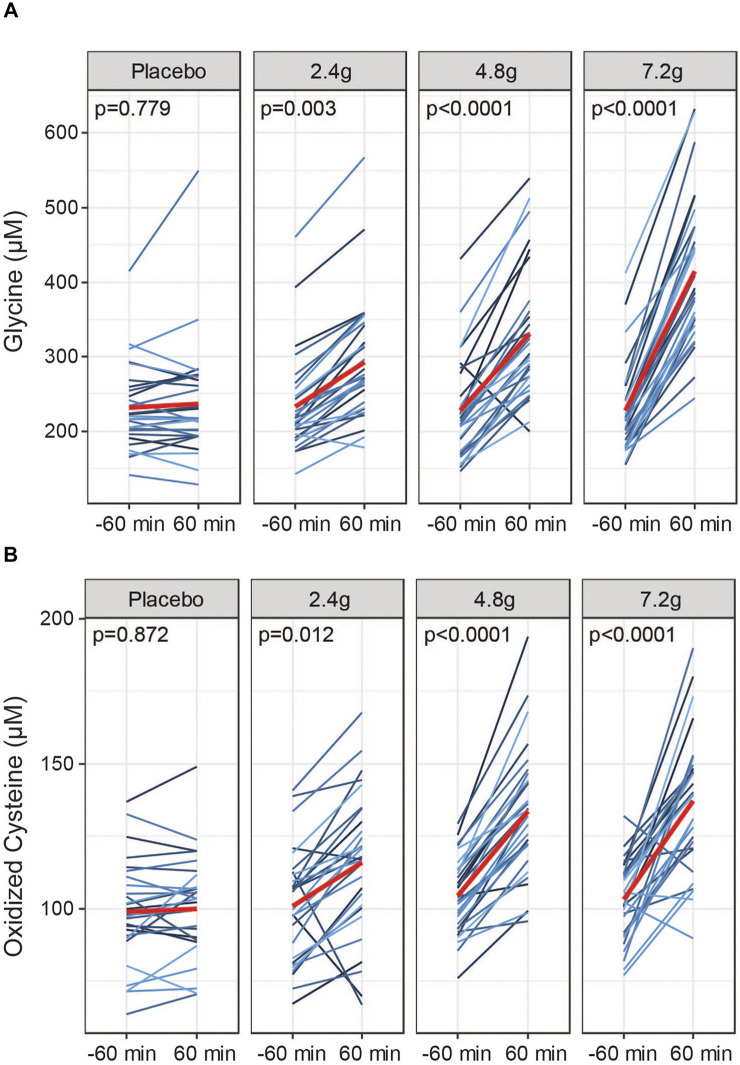
Dosing efficacy of GlyNAC. **(A)** Dose responses to acute oral intake of GlyNAC on glycine and **(B)** oxidized cysteine in plasma of older adults at Visit 2. Values were obtained at −60 min prior and 60 min after consumption of the active doses or placebo. Red lines represent the means. *p* = *p*-values, parametric paired t-tests between pre and post dose.

To compare bioavailability of GlyNAC in blood plasma in response to each dose, we quantified fasting levels of glycine, NAC and oxidized cystine 60 min before and after oral intake. Quantifying the pool of oxidized cysteine can be used as a surrogate endpoint for NAC dosing; acetyl groups are removed from NAC in the gastrointestinal tract and the liver, releasing cysteine into the circulation where it is present in a redox equilibrium between the reduced form, cysteine, and the oxidized form, cystine (Borgstrom and Kagedal, 1990). Measuring oxidized cysteine has the advantage of a higher sensitivity and better practicality in clinical trials using larger cohort sizes to determine the dosing efficacy compared to the direct measurement of reduced cysteine, which is difficult to measure and that can be prone to oxidation artefacts. GlyNAC supplementation led to a dose-dependent increase of circulating levels of glycine in blood plasma measured at 60 min after dose intake at all three doses (Figure 3A). We also detected dose-dependent increases of oxidized cysteine in response to GlyNAC administration (Figure 3B) while total cysteine disulfide levels, which include also protein-bound cysteine, remained unchanged (Supplementary Figure S2A). Thus, oral intake of GlyNAC leads to an efficient and dose-dependent absorption of glycine and NAC within 60 min. In contrast, free NAC is only detectable at the medium and high doses in a subset of subjects (Supplementary Figure S2B). Taken together, glycine and NAC are readily absorbed after oral intake within 1 h at all three active doses.

### Effects of GlyNAC Supplementation on GSH Status

**FIGURE 4:**
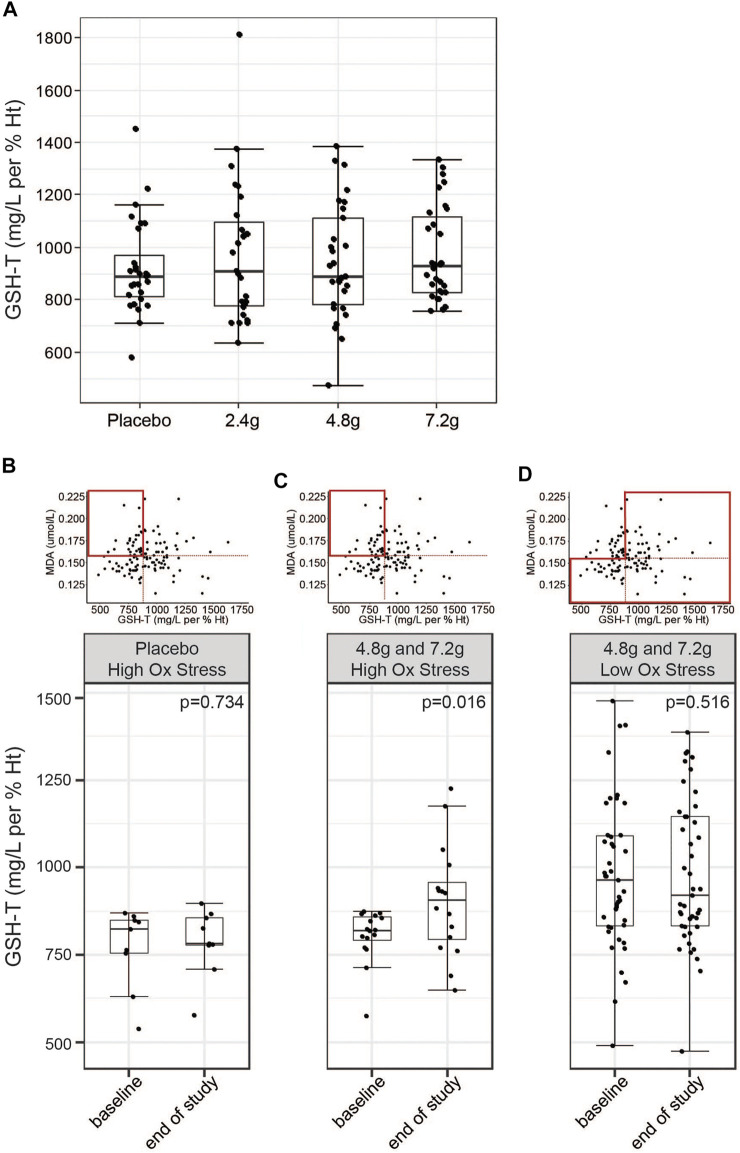
Effects of GlyNAC on circulating levels of GSH-T. **(A)** Effects of daily intake of placebo or three different doses of GlyNAC on GSH-T at Visit 4. Values were measured in whole blood samples in fasted subjects. The last GlyNAC dose was consumed the evening before the measurement. **(B,C)** Post-hoc subset analysis of response to **(B)** placebo or **(C)** GlyNAC treatment (4.8 and 7.2 g groups) in subjects characterized by elevated levels of oxidative stress (MDA above the median) and low baseline glutathione values (GSH-T below the median) in fasted state at Visit 2 and Visit 4. **(D)** Post-hoc subset analysis of response to high doses of GlyNAC treatment (4.8 and 7.2 g) in subjects characterized by low oxidative stress in fasted state at Visit 2 and Visit 4. Boxplots show the median, the first to third quartile, the 1.5x interquartile ranges, and outliers. *p* = *p*-value, nonparametric paired Wilcoxon/Mann-Whitney tests.

The primary endpoint of the study was defined as the difference in GSH-T normalized to hematocrit compared to placebo at the end of the study. No significant differences were observed at any of the three doses tested compared to the placebo treated group (LS-means, placebo = 903.5; 2.4 g = 947.7; 4.8 g = 907.7; 7.2 g = 959.6 mg/L per % Ht, *p*-value 2.4 g *vs.* placebo = 0.9039; *p*-value 4.8 g *vs.* placebo = 0.9340; *p*-value 7.2 g *vs.* placebo = 0.2778) and no significant intra-individual increases were detected comparing start of the study to the end of the study (Figure 4A; Table 2). Similarly, GlyNAC supplementation also did not change significantly the ratio of GSH-F:GSSG (LS-means, placebo = 12.49; 2.4 g = 13.53; 4.8 g = 11.34; 7.2 g = 12.65 mg/L per % Ht, *p*-value 2.4 g *vs.* placebo = 0.276; *p*-value 4.8 g *vs.* placebo = 0.502; *p*-value 7.2 g *vs.* placebo = 0.739) (Table 2). Levels of MDA or total cysteine were not impacted by GlyNAC supplementation compared to placebo, although a moderate but significant intra-individual lowering of total cysteine levels was observed at the highest dose (means, 7.2 g, start of study = 415.9, end of study = 396.7 µmol/L, *p*-value = 0.028) (Supplementary Table S4).

**TABLE 2:** Effects of GlyNAC supplementation on whole blood glutathione levels. Acute and chronic changes in total glutathione (GSH-T) and reduced to oxidized glutathione (GSH-F:GSSG) in whole blood.

	Baseline			End of study			*P* ^*c*^	*P* ^*e*^
*Dose*	*Pre-dose*	*Post-dose*	*P* ^*a*^	*Pre-dose*	*Post-dose*	*P* ^*a*^		
*GSH-T*								
Placebo	886.8 (819.9, 959.1)	878.9 (812.7, 950.6)	0.678	903.5 (835.4, 977.2)	925.7 (855.9, 1001.1)	0.443	0.528	-
2.4 g	892.8 (826.6, 964.3)	892.9 (826.7, 964.4)	0.896	947.7 (876.6, 1014.6)	921.2 (852.1, 996)	0.182	0.045	0.904
4.8 g	874.7 (810.9, 943.5)	838.7 (777.7, 904.8)	0.106	907.7 (841.1, 979.5)	925.7 (857.8, 998.9)	0.685	0.200	0.934
7.2 g	927.7 (860.1, 1000.7)	922.1 (854.8, 994.6)	0.866	959.6 (889.7, 1035.1)	937.8 (869, 1011.9)	0.380	0.237	0.278
*GSH-F*								
Placebo	713.7 (649.0, 784.9)	717.3 (652.5, 788.4)	0.904	739.8 (673.0, 813.2)	760.8 (692.2, 836.3)	0.434	0.279	-
2.4 g	725.5 (661.1, 796.1)	743.2 (677.2, 815.6)	0.428	787.1 (716.5, 864.7)	772.6 (702.8, 848.1)	0.301	0.014	0.361
4.8 g	695.1 (634.4, 761.6)	684.4 (624.6, 749.8)	0.372	736.7 (672.1, 807.5)	768.5 (701.1, 842.4)	0.284	0.070	0.950
7.2 g	745.9 (680.8, 817.3)	758.1 (691.9, 830.7)	0.609	788.5 (719.7, 864.0)	777.9 (709.4, 853.1)	0.654	0.080	0.512
*GSH-F:GSSG*								
Placebo	10.68 (8.88, 12.85)	11.95 (9.94, 14.36)	0.645	12.49 (10.39, 15.01)	12.38 (10.3, 14.87)	0.674	0.040	-
2.4 g	11.79 (9.84, 14.19)	13.6 (11.35, 16.29)	0.510	13.53 (11.26, 16.26)	14.29 (11.9, 17.17)	0.765	0.068	0.276
4.8 g	10.17 (8.52, 12.15)	12.15 (10.18, 14.52)	0.274	11.34 (9.49, 13.57)	13.51 (11.3, 16.15)	0.157	0.138	0.502
7.2 g	11.06 (9.26, 13.21)	12.75 (10.67, 15.22)	0.462	12.65 (10.59, 15.1)	12.93 (10.8, 15.48)	0.941	0.065	0.739

Values for GSH-T and GSH-F:GSSG were normalized to hematocrit and expressed as LS-Means in mg/L per % of hematocrit with 95% Confidence Interval. GSH-T, total glutathione; GSH-F, free reduced glutathione; GSSG, oxidized glutathione; *P* ^*a*^ *= p-value pre-dose vs. post-dose, P* ^*c*^ *= p-value for change from baseline to end of study at pre-dose, P* ^*e*^ *= p-value end of study at pre-dose comparing placebo vs. active dose group*.

We next asked the question whether glutathione biosynthesis from dietary glycine and cysteine is regulated by cellular glutathione demand. To this end, we performed a post hoc analysis on a subset of the study population that is characterized by increased oxidative stress, as determined by the values above the median for MDA, while showing low levels of baseline glutathione levels, as characterized by values below the median. Subjects from the placebo group did not show differences in total glutathione levels when comparing start with end of the study (medians, V2 = 824.4, V4 = 780 mg/L per % Ht, *p*-value = 0.734) (Figure 4B). In contrast, pooled analysis of subjects from the 4.8 and 7.2 g treatment groups showed a 10.47% increase of total glutathione levels at the end of the 2 weeks of GlyNAC supplementation (medians, V2 = 819.7, V4 = 905.4 mg/L per %Ht, *p*-value = 0.016) (Figure 4C). The same analysis but combining the subjects from the 4.8 and 7.2 g groups that are not characterized by high oxidative stress did not show changes in GSH-T (medians, V2 = 947.2, V4 = 920.7 mg/L per %Ht, *p*-value = 0.516) (Figure 4D). An analysis based on elevated total cysteine (above the median) and low GSH-T (below the median) showed a similar result on GlyNAC efficacy in this subpopulation (medians, V2 = 817.7, V4 = 859.3 mg/L per %Ht, *p*-value = 0.015) (Supplementary Figure S3). These data suggest that healthy older adults with GSH-T levels that are similar to those of young adults may not utilize intra-cellular GlyNAC to generate additional GSH-T, while a subset of subjects with higher glutathione demand may be responding.

### Glycine Levels are Increased in Response to GlyNAC Independently of GSH Status

An important observation of this study is the lower levels of glycine in healthy older adults (by approximately 15.5% in plasma and 8.9% in whole blood) compared to young subjects (Table 1). We therefore asked the question whether fasting glycine status is related to markers of glutathione status or oxidative stress. However, no significant associations of circulating glycine concentrations with levels of GSH-T, GSH-F:GSSG, or MDA were detected (Supplementary Figures S4A−C). A significant but moderate association of lower levels of glycine with higher levels of total cysteine could be detected (Supplementary Figure S4D). In contrast, older adults from this cohort showed a negative correlation between glycine status and the Homeostatic Model Assessment for Insulin Resistance (HOMA-IR), a composite score indicating differences in insulin sensitivity (Supplementary Figure S4E). This observation in older adults is consistent with previous studies that report associations of low glycine status with elevated insulin resistance (Newgard et al., 2009; Wittemans et al., 2019).

**FIGURE 5:**
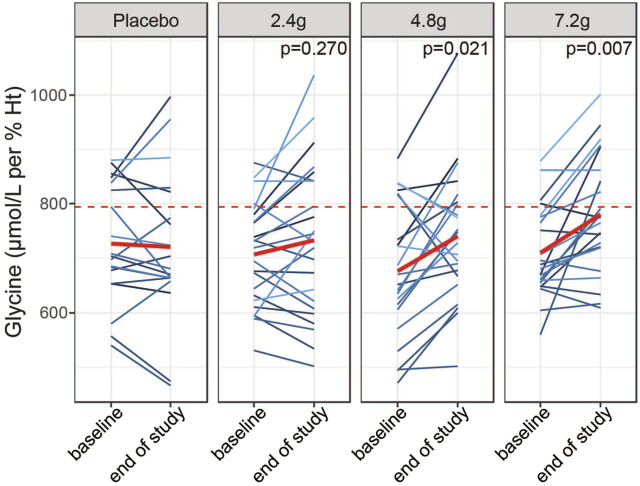
Dose dependent effects of GlyNAC supplementation on glycine status. Effects of daily intake of placebo or different doses of GlyNAC on whole blood glycine levels in a subpopulation of older adults (excluding the group above the third quartile). Glycine levels have been compared before (baseline, Visit 2) and after the 2-weeks of treatment (end of study, Visit 4) in samples taken prior to the dosing with GlyNAC (−60 min) The red dashed lines represent the median of the young adults. *p* = *p*-value, linear mixed model testing intra-individual changes from baseline to end of study within each dose group and from placebo group.

We next asked whether 2 weeks of daily supplementation with GlyNAC increased fasting levels of glycine towards that of young subjects. To this end, we compared glycine levels at the end of the study between the four treatment groups while adjusting for baseline glycine concentrations. We excluded the group above the third quartile of glycine levels to remove subjects that may not have adhered to the overnight fast or that have particularly high fasting glycine levels due to other reasons. We found that the medium and high doses efficiently increased glycine levels compared to baseline (4.8 g coefficient = 65.10, *p*-value = 0.021; 7.2 g coefficient = 74.9, *p*-value = 0.007, linear model ANCOVA, reference level: placebo), whereas the 2.4 g groups did not show a significant effect (2.4 g, coefficient = 29.99, *p*-value = 0.27) (Figure 5; Supplementary Tables S5, S6). Of note, the medium and high doses were able to increase glycine levels close to the median level observed in young individuals (Figure 5, red dashed line). Changes in blood plasma in response to GlyNAC supplementation were not significant, although the medium dose showed a trend towards improved glycine status (4.8 g, *p*-value = 0.068) (Supplementary Figure S5). In summary, these results suggest that lower fasting glycine values in older adults may be efficiently restored towards intracellular blood levels of young adults within 2 weeks of supplementation.

## Discussion

Recruitment to this study followed stringent criteria to select for healthy volunteers. We provide evidence that even in this cohort representing healthy aging, several markers related to oxidative damage differ significantly from that of young adults, suggesting a pro-oxidative systemic environment. Consistent with a measurable impact of the pro-oxidative cellular status on the GSH system, levels of the oxidized dimer GSSG were elevated compared to that of young subjects. However, the ratio of GSH-F:GSSG was only moderately affected, and GSH-F or GSH-T levels did not differ. Further, we did not detect a correlation of GSH status and levels of MDA or total cysteine, suggesting that the GSH buffer capacity is not a limiting factor for protection from oxidative stress in this cohort of healthy older adults.

Consistent with the lack of differences of GSH levels between young and older adults, GlyNAC supplementation did not increase GSH-T or the GSH-F:GSSG ratio. GSH biosynthesis is proposed to be regulated as a rheostat system that is designed to maintain sufficient antioxidant capacities, while preventing an overproduction of low molecular antioxidants that may interfere with the essential roles of ROS signaling (Ristow et al., 2009; Ristow, 2014; Shadel and Horvath, 2015). To test the hypothesis that glutathione restoration occurs on the basis of cellular demand, we performed a post hoc analysis in participants that showed low levels of GSH-T (below the median) and high levels of MDA (above the median). Indeed, these subjects responded to GlyNAC supplementation with increases of GSH levels when we pooled the 4.8 and 7.2 g intervention groups. Similar results were obtained when total cysteine was used as marker of elevated oxidative stress. Of note, the proportion of individuals that increased GSH levels based on these criteria was 27.5%, which would represent a substantial proportion of the healthy older population.

Beyond the established benefits of NAC in some disorders, several trials in other disease conditions have shown inconsistent efficacy or no efficacy of GSH supplementation on outcomes, and the causal relationship of glutathione depletion to disease progression remains incompletely understood (van Zandwijk et al., 2000; Rushworth and Megson, 2014; Szkudlinska et al., 2016; Weisbord et al., 2018). In our study, blood GSH levels and the GSH-F:GSSG ratio show a high variability including many subjects that have comparable levels to those of young subjects, which can only be partially explained by technical variability, and more likely is due to interindividual differences in genetics and environmental factors. In fact, some subjects may cope well with oxidative stress through GSH-GSSG buffering and a stratification of patients for GSH demand could be a promising strategy to identify those that may benefit most from NAC or GlyNAC supplementation. Further studies are warranted to confirm this hypothesis and to understand the effects of GlyNAC supplementation in subjects with elevated oxidative stress and low baseline GSH status. In addition, investigating GlyNAC effects on GSH regulation in tissues would be of interest; we cannot exclude that GSH levels in tissues other than blood cells were more severely affected by oxidative stress since erythrocytes lack mitochondria and therefore the primary source of ROS generation. Finally, including additional biomarkers in blood and urine could help to detect more subtle differences in oxidative damage in generally healthy subjects. For example, F2-Isoprostanes, a class of lipid peroxides, are considered more stable and therefore more reliable indicators of oxidative stress than malondialdehyde and total cysteine disulfide used in this study (Milne et al., 2007; Ho et al., 2013).

Our data add to a body of evidence that healthy aging is accompanied by elevated markers of cardiovascular risk, which included significantly increased levels of CRP, HOMA-IR, triglycerides, and cholesterol (Peng et al., 2019). Further, the concentration of the oxidative damage marker total cysteine disulfide of >300 uM in older adults is within the range of what has been described as being associated with elevated cardiovascular risk compared to that of young subjects (El-Khairy et al., 2001). A group of elderly people (mean age = 70 years) described by Sekhar et al. to have severe GSH depletion (GSH-F:GSSG ratio = 7.4 in old *vs.* 18.9 in young) was reported to have elevated insulin resistance (HOMA-IR of 4 in older adults compared to 1.6 in young subjects, *p* < 0.01, *n* = 8 each) (Sekhar et al., 2011; Nguyen et al., 2013). A second recently published cohort also showed severe GSH depletion in older subjects (Ratio of GSH-F:GSSG of 1.1 in older subjects compared to 10.1 in young subjects, *p*-value = 0.0003, *n* = 8 each group) that were accompanied by high levels of insulin resistance (HOMA-IR of 11.4 in older adults compared to 1.7 in young subjects, *p*-value<0.0001) and chronic systemic inflammation (CRP: young adults = 2.4 mg/L; older adults = 4.8 mg/L, *p*-value < 0.0001) (Kumar et al., 2021a). Ethnicity and lifestyle factors due to the geographic location of the respective participants may explain differences in GSH redox status among the cohorts. Nevertheless, differences in cardiometabolic health status between the previous studies by Sekhar et al. and the cohort of older adults investigated in this study may be a cause of the exhaustion of the GSH system. Whether supplementation of GlyNAC improves cardiometabolic health, for example in older adults with pronounced insulin resistance or high inflammation status remains to be studied.

Alternatively, cardiometabolic health may impact glycine status independently of GSH redox status. We found glycine levels to be significantly lower in older adults compared to that of young subjects and to be inversely correlated with insulin resistance; glycine levels were 15.5% lower in plasma, an extent that is similar to what has been described in obese, insulin-resistant subjects (Newgard et al., 2009). The significantly lower glycine status in healthy older adults is surprising as glycine is a non-essential amino acid whose levels are regulated by *de novo* synthesis and dietary intake. A regular Western Diet provides about 3 g of glycine per day while the endogenous synthesis rate is estimated to generate approximately 1.5–3 g per day (Melendez-Hevia et al., 2009). The lower fasting glycine levels that are negatively correlated with HOMA-IR point towards a link of glycine status and cardiometabolic health in healthy aging, similar to what has been reported in people with diabetes (Wittemans et al., 2019). Consistent with this concept, an intervention trial with 15 g of glycine daily during 3 months lowered HbA_1c_ levels in people with diabetes (Cruz et al., 2008), while acute ingestion of glycine together with an oral glucose bolus lowered glucose excursions (Gannon et al., 2002). More studies are warranted to identify target populations that may benefit from supplementary dietary glycine intake.

In summary, this study suggests that GlyNAC supplementation is safe and well tolerated in older adults and may support GSH biosynthesis in individuals with increased oxidative stress and compromised GSH stores. In individuals without an increased oxidative burden, circulating GSH remained stable.

## Data Availability

The raw data supporting the conclusion of this article will be made available by the authors, without undue reservation.

## References

[B1] Beckman K. B. Ames B. N. (1998). The Free Radical Theory of Aging Matures. Physiol. Rev. 78, 547–581. 10.1152/physrev.1998.78.2.547 9562038

[B2] Borgström L. Kågedal B. (1990). Dose Dependent Pharmacokinetics ofN-ACETYLCYSTEINE AFTER ORAL DOSING TO MAN. Biopharm. Drug Dispos. 11, 131–136. 10.1002/bdd.2510110205 2328298

[B3] Butterworth C. E. Jr. Santini R. Jr. Perez-Santiago E. (1958). The Absorption of glycine and its Conversion to Serine in Patients with Sprue. J. Clin. Invest. 37, 20–27. 10.1172/jci103580 13491708 PMC293052

[B4] Cruz M. Maldonado-Bernal C. Mondragón-Gonzalez R. Sanchez-Barrera R. Wacher N. H. Carvajal-Sandoval G. (2008). Glycine Treatment Decreases Proinflammatory Cytokines and Increases Interferon-γ in Patients with Type 2 Diabetes. J. Endocrinol. Invest. 31, 694–699. 10.1007/bf03346417 18852529

[B5] De Rosa S. C. Zaretsky M. D. Dubs J. G. Roederer M. Anderson M. Green A. (2000). N-acetylcysteine Replenishes Glutathione in HIV Infection. Eur. J. Clin. Invest. 30, 915–929. 10.1046/j.1365-2362.2000.00736.x 11029607

[B6] El-Khairy L. Ueland P. M. Refsum H. Graham I. M. Vollset S. E. European Concerted Action P. (2001). Plasma Total Cysteine as a Risk Factor for Vascular Disease: The European Concerted Action Project. Circulation 103, 2544–2549. 10.1161/01.cir.103.21.2544 11382721

[B7] Forman H. J. Zhang H. Rinna A. (2009). Glutathione: Overview of its Protective Roles, Measurement, and Biosynthesis. Mol. Aspects Med. 30, 1–12. 10.1016/j.mam.2008.08.006 18796312 PMC2696075

[B8] Forrester S. J. Kikuchi D. S. Hernandes M. S. Xu Q. Griendling K. K. (2018). Reactive Oxygen Species in Metabolic and Inflammatory Signaling. Circ. Res. 122, 877–902. 10.1161/circresaha.117.311401 29700084 PMC5926825

[B9] Fu X. Cate S. A. Dominguez M. Osborn W. Özpolat T. Konkle B. A. (2019). Cysteine Disulfides (Cys-Ss-X) as Sensitive Plasma Biomarkers of Oxidative Stress. Sci. Rep. 9, 115. 10.1038/s41598-018-35566-2 30643157 PMC6331564

[B10] Gannon M. C. Nuttall J. A. Nuttall F. Q. (2002). The Metabolic Response to Ingested glycine. Am. J. Clin. Nutr. 76, 1302–1307. 10.1093/ajcn/76.6.1302 12450897

[B11] García-Prat L. Martínez-Vicente M. Perdiguero E. Ortet L. Rodríguez-Ubreva J. Rebollo E. (2016). Autophagy Maintains Stemness by Preventing Senescence. Nature 529, 37–42. 10.1038/nature16187 26738589

[B12] Gupta S. K. Kamendulis L. M. Clauss M. A. Liu Z. (2016). A Randomized, Placebo-Controlled Pilot Trial of N-Acetylcysteine on Oxidative Stress and Endothelial Function in HIV-Infected Older Adults Receiving Antiretroviral Treatment. AIDS 30, 2389–2391. 10.1097/qad.0000000000001222 27603163 PMC5015649

[B13] Hamanishi T. Furuta H. Kato H. Doi A. Tamai M. Shimomura H. (2004). Functional Variants in the Glutathione Peroxidase-1 (GPx-1) Gene Are Associated with Increased Intima-media Thickness of Carotid Arteries and Risk of Macrovascular Diseases in Japanese Type 2 Diabetic Patients. Diabetes 53, 2455–2460. 10.2337/diabetes.53.9.2455 15331559

[B14] Hargreaves I. P. Sheena Y. Land J. M. Heales S. J. R. (2005). Glutathione Deficiency in Patients with Mitochondrial Disease: Implications for Pathogenesis and Treatment. J. Inherit. Metab. Dis. 28, 81–88. 10.1007/s10545-005-4160-1 15702408

[B15] Harman D. (1992). Free Radical Theory of Aging. Mutat. Research/DNAging 275, 257–266. 10.1016/0921-8734(92)90030-s 1383768

[B16] Herzenberg L. A. De Rosa S. C. Dubs J. G. Roederer M. Anderson M. T. Ela S. W. (1997). Glutathione Deficiency Is Associated with Impaired Survival in HIV Disease. Proc. Natl. Acad. Sci. 94, 1967–1972. 10.1073/pnas.94.5.1967 9050888 PMC20026

[B17] Hildebrandt W. Sauer R. Bonaterra G. Dugi K. A. Edler L. Kinscherf R. (2015). Oral N-Acetylcysteine Reduces Plasma Homocysteine Concentrations Regardless of Lipid or Smoking Status. Am. J. Clin. Nutr. 102, 1014–1024. 10.3945/ajcn.114.101964 26447155

[B18] Hirai D. M. Jones J. H. Zelt J. T. Da Silva M. L. Bentley R. F. Edgett B. A. (2017). Oral N-Acetylcysteine and Exercise Tolerance in Mild Chronic Obstructive Pulmonary Disease. J. Appl. Physiol. (1985) 122, 1351–1361. 10.1152/japplphysiol.00990.2016 28255088

[B19] Ho E. Karimi Galougahi K. Liu C.-C. Bhindi R. Figtree G. A. (2013). Biological Markers of Oxidative Stress: Applications to Cardiovascular Research and Practice. Redox Biol. 1, 483–491. 10.1016/j.redox.2013.07.006 24251116 PMC3830063

[B20] Kumar P. Liu C. Suliburk J. W. Minard C. G. Muthupillai R. Chacko S. (2020). Supplementing Glycine and N-Acetylcysteine (GlyNAC) in Aging HIV Patients Improves Oxidative Stress, Mitochondrial Dysfunction, Inflammation, Endothelial Dysfunction, Insulin Resistance, Genotoxicity, Strength, and Cognition: Results of an Open-Label Clinical Trial. Biomedicines 8, 390. 10.3390/biomedicines8100390 PMC7601820 33007928

[B21] Kumar P. Liu C. Hsu J. W. Chacko S. Minard C. Jahoor F. (2021a). Glycine and N-Acetylcysteine (GlyNAC) Supplementation in Older Adults Improves Glutathione Deficiency, Oxidative Stress, Mitochondrial Dysfunction, Inflammation, Insulin Resistance, Endothelial Dysfunction, Genotoxicity, Muscle Strength, and Cognition: Results of a Pilot Clinical Trial. Clin. Transl Med. 11, e372. 10.1002/ctm2.372 33783984 PMC8002905

[B22] Kumar P. Osahon O. Vides D. B. Hanania N. Minard C. G. Sekhar R. V. (2021b). Severe Glutathione Deficiency, Oxidative Stress and Oxidant Damage in Adults Hospitalized with COVID-19: Implications for GlyNAC (Glycine and N-Acetylcysteine) Supplementation. Antioxidants (Basel) 11, 50. 10.3390/antiox11010050 35052554 PMC8773164

[B23] Lang C. A. Naryshkin S. Schneider D. L. Mills B. J. Lindeman R. D. (1992). Low Blood Glutathione Levels in Healthy Aging Adults. J. Lab. Clin. Med. 120, 720–725. 1431500

[B24] Lang C. A. Mills B. J. Mastropaolo W. Liu M. C. (2000). Blood Glutathione Decreases in Chronic Diseases. J. Lab. Clin. Med. 135, 402–405. 10.1067/mlc.2000.105977 10811055

[B25] Lauterburg B. H. Corcoran G. B. Mitchell J. R. (1983). Mechanism of Action of N-Acetylcysteine in the protection against the Hepatotoxicity of Acetaminophen in Rats *In Vivo* . J. Clin. Invest. 71, 980–991. 10.1172/jci110853 6833497 PMC436956

[B26] Lee J. Giordano S. Zhang J. (2012). Autophagy, Mitochondria and Oxidative Stress: Cross-Talk and Redox Signalling. Biochem. J. 441, 523–540. 10.1042/bj20111451 22187934 PMC3258656

[B27] Malmezat T. Breuille D. Capitan P. Mirand P. P. Obled C. (2000). Glutathione Turnover Is Increased during the Acute Phase of Sepsis in Rats. J. Nutr. 130, 1239–1246. 10.1093/jn/130.5.1239 10801925

[B28] Martina V. Masha A. Gigliardi V. R. Brocato L. Manzato E. Berchio A. (2008). Long-term N-Acetylcysteine and L-Arginine Administration Reduces Endothelial Activation and Systolic Blood Pressure in Hypertensive Patients with Type 2 Diabetes. Diabetes Care 31, 940–944. 10.2337/dc07-2251 18268065

[B29] Meléndez-Hevia E. De Paz-Lugo P. Cornish-Bowden A. Cárdenas M. L. (2009). A Weak Link in Metabolism: the Metabolic Capacity for glycine Biosynthesis Does Not Satisfy the Need for Collagen Synthesis. J. Biosci. 34, 853–872. 10.1007/s12038-009-0100-9 20093739

[B30] Milne G. L. Sanchez S. C. Musiek E. S. Morrow J. D. (2007). Quantification of F2-Isoprostanes as a Biomarker of Oxidative Stress. Nat. Protoc. 2, 221–226. 10.1038/nprot.2006.375 17401357

[B31] Newgard C. B. An J. Bain J. R. Muehlbauer M. J. Stevens R. D. Lien L. F. (2009). A Branched-Chain Amino Acid-Related Metabolic Signature that Differentiates Obese and Lean Humans and Contributes to Insulin Resistance. Cel Metab. 9, 311–326. 10.1016/j.cmet.2009.02.002 PMC3640280 19356713

[B32] Nguyen D. Samson S. L. Reddy V. T. Gonzalez E. V. Sekhar R. V. (2013). Impaired Mitochondrial Fatty Acid Oxidation and Insulin Resistance in Aging: Novel Protective Role of Glutathione. Aging Cell 12, 415–425. 10.1111/acel.12073 23534396

[B33] Nguyen D. Hsu J. W. Jahoor F. Sekhar R. V. (2014). Effect of Increasing Glutathione with Cysteine and glycine Supplementation on Mitochondrial Fuel Oxidation, Insulin Sensitivity, and Body Composition in Older HIV-Infected Patients. J. Clin. Endocrinol. Metab. 99, 169–177. 10.1210/jc.2013-2376 24081740 PMC3879663

[B34] Peng P.-S. Kao T.-W. Chang P.-K. Chen W.-L. Peng P.-J. Wu L.-W. (2019). Association between HOMA-IR and Frailty Among U.S. Middle-Aged and Elderly Population. Sci. Rep. 9, 4238. 10.1038/s41598-019-40902-1 30862906 PMC6414687

[B35] Redman L. M. Smith S. R. Burton J. H. Martin C. K. Il'yasova D. Ravussin E. (2018). Metabolic Slowing and Reduced Oxidative Damage with Sustained Caloric Restriction Support the Rate of Living and Oxidative Damage Theories of Aging. Cel Metab. 27, 805–815.e4. 10.1016/j.cmet.2018.02.019 PMC5886711 29576535

[B36] Ristow M. Zarse K. Oberbach A. Kloting N. Birringer M. Kiehntopf M. (2009). Antioxidants Prevent Health-Promoting Effects of Physical Exercise in Humans. Proc. Natl. Acad. Sci. 106, 8665–8670. 10.1073/pnas.0903485106 19433800 PMC2680430

[B37] Ristow M. (2014). Unraveling the Truth about Antioxidants: Mitohormesis Explains ROS-Induced Health Benefits. Nat. Med. 20, 709–711. 10.1038/nm.3624 24999941

[B38] Roum J. H. Buhl R. Mcelvaney N. G. Borok Z. Crystal R. G. (1993). Systemic Deficiency of Glutathione in Cystic Fibrosis. J. Appl. Physiol. (1985) 75, 2419–2424. 10.1152/jappl.1993.75.6.2419 8125859

[B39] Rushworth G. F. Megson I. L. (2014). Existing and Potential Therapeutic Uses for N-Acetylcysteine: the Need for Conversion to Intracellular Glutathione for Antioxidant Benefits. Pharmacol. Ther. 141, 150–159. 10.1016/j.pharmthera.2013.09.006 24080471

[B40] Samiec P. S. Drews-Botsch C. Flagg E. W. Kurtz J. C. Sternberg P. Jr. Reed R. L. (1998). Glutathione in Human Plasma: Decline in Association with Aging, Age-Related Macular Degeneration, and Diabetes. Free Radic. Biol. Med. 24, 699–704. 10.1016/s0891-5849(97)00286-4 9586798

[B41] Sekhar R. V. Patel S. G. Guthikonda A. P. Reid M. Balasubramanyam A. Taffet G. E. (2011). Deficient Synthesis of Glutathione Underlies Oxidative Stress in Aging and Can Be Corrected by Dietary Cysteine and glycine Supplementation. Am. J. Clin. Nutr. 94, 847–853. 10.3945/ajcn.110.003483 21795440 PMC3155927

[B42] Shadel G. S. Horvath T. L. (2015). Mitochondrial ROS Signaling in Organismal Homeostasis. Cell 163, 560–569. 10.1016/j.cell.2015.10.001 26496603 PMC4634671

[B43] Sies H. (1997). Oxidative Stress: Oxidants and Antioxidants. Exp. Physiol. 82, 291–295. 10.1113/expphysiol.1997.sp004024 9129943

[B44] Sies H. (1999). Glutathione and its Role in Cellular Functions. Free Radic. Biol. Med. 27, 916–921. 10.1016/s0891-5849(99)00177-x 10569624

[B45] Sies H. (2015). Oxidative Stress: a Concept in Redox Biology and Medicine. Redox Biol. 4, 180–183. 10.1016/j.redox.2015.01.002 25588755 PMC4309861

[B46] Smilkstein M. J. Knapp G. L. Kulig K. W. Rumack B. H. (1988). Efficacy of Oral N-Acetylcysteine in the Treatment of Acetaminophen Overdose. Analysis of the National Multicenter Study (1976 to 1985). N. Engl. J. Med. 319, 1557–1562. 10.1056/nejm198812153192401 3059186

[B47] Szkudlinska M. A. Von Frankenberg A. D. Utzschneider K. M. (2016). The Antioxidant N-Acetylcysteine Does Not Improve Glucose Tolerance or β-cell Function in Type 2 Diabetes. J. Diabetes its Complications 30, 618–622. 10.1016/j.jdiacomp.2016.02.003 PMC4834245 26922582

[B48] Townsend D. M. Tew K. D. Tapiero H. (2003). The Importance of Glutathione in Human Disease. Biomed. Pharmacother. 57, 145–155. 10.1016/s0753-3322(03)00043-x 12818476 PMC6522248

[B49] van Zandwijk N. Dalesio O. Pastorino U. De Vries N. Van Tinteren H. (2000). EUROSCAN, a Randomized Trial of Vitamin A and N-Acetylcysteine in Patients with Head and Neck Cancer or Lung Cancer. For the EUropean Organization for Research and Treatment of Cancer Head and Neck and Lung Cancer Cooperative Groups. J. Natl. Cancer Inst. 92, 977–986. 10.1093/jnci/92.12.977 10861309

[B50] Weisbord S. D. Gallagher M. Jneid H. Garcia S. Cass A. Thwin S.-S. (2018). Outcomes after Angiography with Sodium Bicarbonate and Acetylcysteine. N. Engl. J. Med. 378, 603–614. 10.1056/nejmoa1710933 29130810

[B51] Wittemans L. B. L. Lotta L. A. Oliver-Williams C. Stewart I. D. Surendran P. Karthikeyan S. (2019). Assessing the Causal Association of glycine with Risk of Cardio-Metabolic Diseases. Nat. Commun. 10, 1060. 10.1038/s41467-019-08936-1 30837465 PMC6400990

[B52] Wu G. Fang Y.-Z. Yang S. Lupton J. R. Turner N. D. (2004). Glutathione Metabolism and its Implications for Health. J. Nutr. 134, 489–492. 10.1093/jn/134.3.489 14988435

[B53] Zhang J. X. Wang Z. M. Zhang J. J. Zhu L. L. Gao X. F. Chen S. L. (2014). Association of Glutathione Peroxidase-1 (GPx-1) Rs1050450 Pro198Leu and Pro197Leu Polymorphisms with Cardiovascular Risk: a Meta-Analysis of Observational Studies. J. Geriatr. Cardiol. 11, 141–150. 10.3969/j.issn.1671-5411.2014.02.003 25009565 PMC4076455

[B54] Zheng J.-P. Wen F.-Q. Bai C.-X. Wan H.-Y. Kang J. Chen P. (2014). Twice Daily N-Acetylcysteine 600 Mg for Exacerbations of Chronic Obstructive Pulmonary Disease (PANTHEON): a Randomised, Double-Blind Placebo-Controlled Trial. Lancet Respir. Med. 2, 187–194. 10.1016/s2213-2600(13)70286-8 24621680

