# Supplementary material for: A Randomized Controlled Clinical Trial in Healthy Older Adults to Determine Efficacy of Glycine and N-Acetylcysteine Supplementation on Glutathione Redox Status and Oxidative Damage
Source: Front Aging. 2022 Mar 7;3:852569. doi: 10.3389/fragi.2022.852569 (PMC9261343; doi:10.3389/fragi.2022.852569)
Supplement: Supplementary file 1 [file DataSheet1.PDF]

SUPPLEMENTARY MATERIAL

Lizzo et al.

1 Supplementary Figures

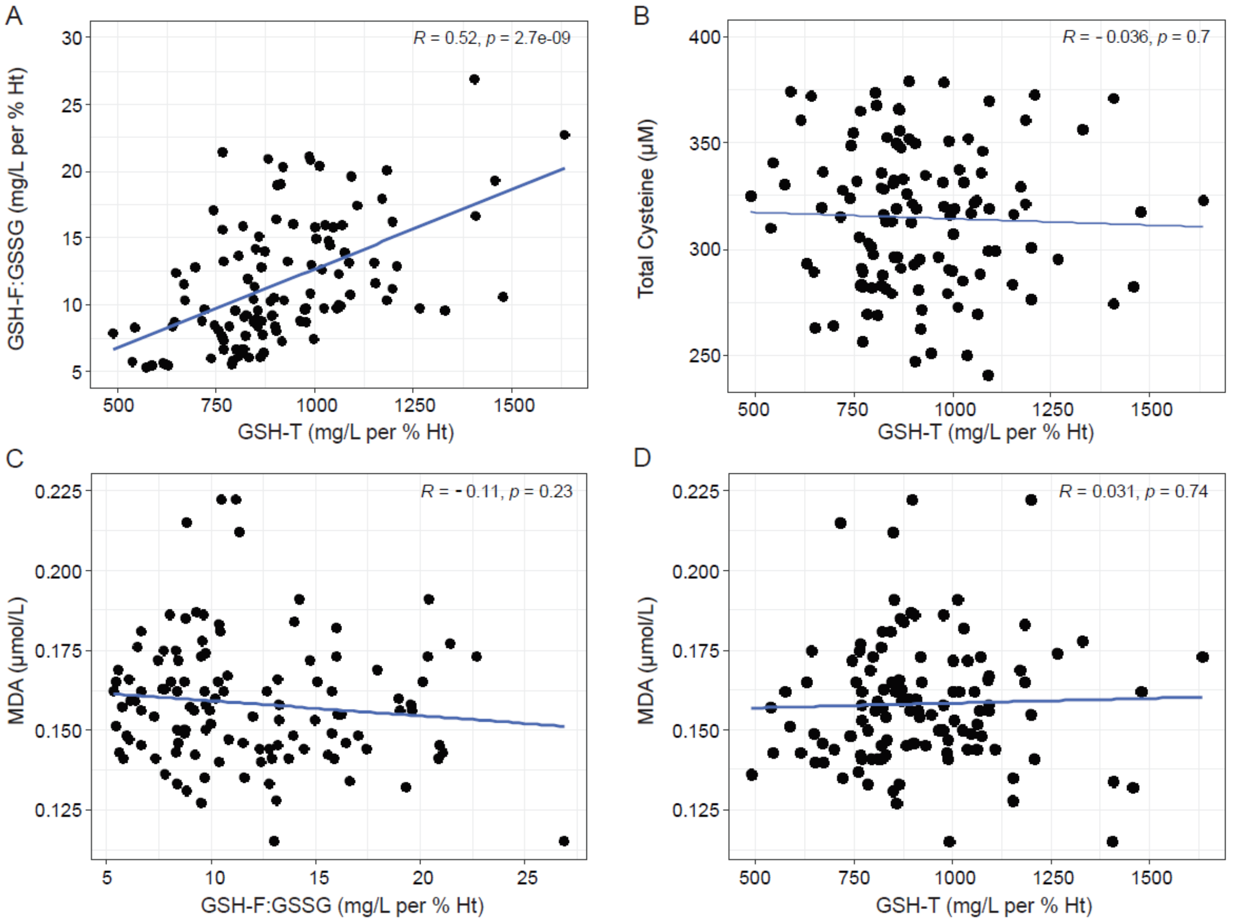

Supplementary Figure 1 Correlation of basal parameters of oxidative stress in older adults.

Correlation between basal parameters of oxidative stress in healthy older adults cohort, comparing GSH-T with GSH-F:GSSG **(A)** and total cysteine **(B)**, and MDA with GSH-F:GSSG **(C)** and GSH-T **(D)**. R= Pearson's linear coefficient, p= p-value.

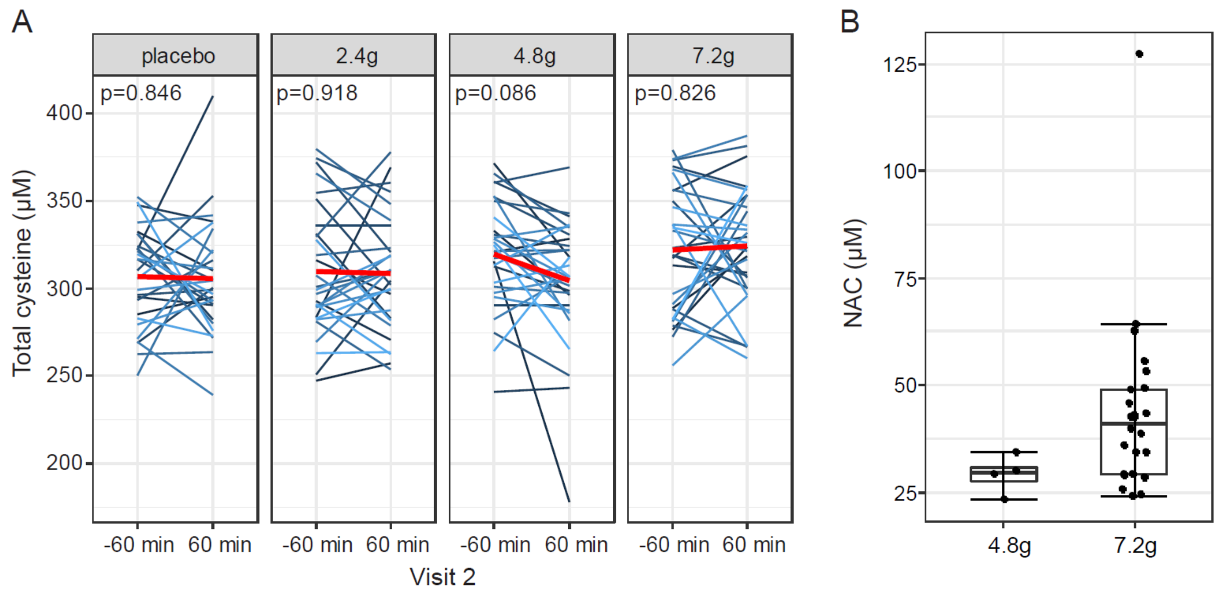

Supplementary Figure 2 Levels of total cysteine and n-acetylcysteine in plasma after product intake

(A) Dose response to acute oral intake of total cysteine in plasma of older adults at Visit 2. Values were obtained at -60min prior and 60min after consumption of the active doses of placebo. Red lines represent the means. **(B)** Levels of the cysteine precursor NAC detected in plasma of study participants at 60 min after ingestion. NAC was not detectable in placebo control subjects or patients that received the 2.4g dose. NAC was detected in a subset of subjects that received the 4.8g and 7.2 g doses. Statistical analysis was not performed due to the incomplete dataset. Boxplot shows the median, the first to third quartile, the 1.5x interquartile ranges, and outliers.

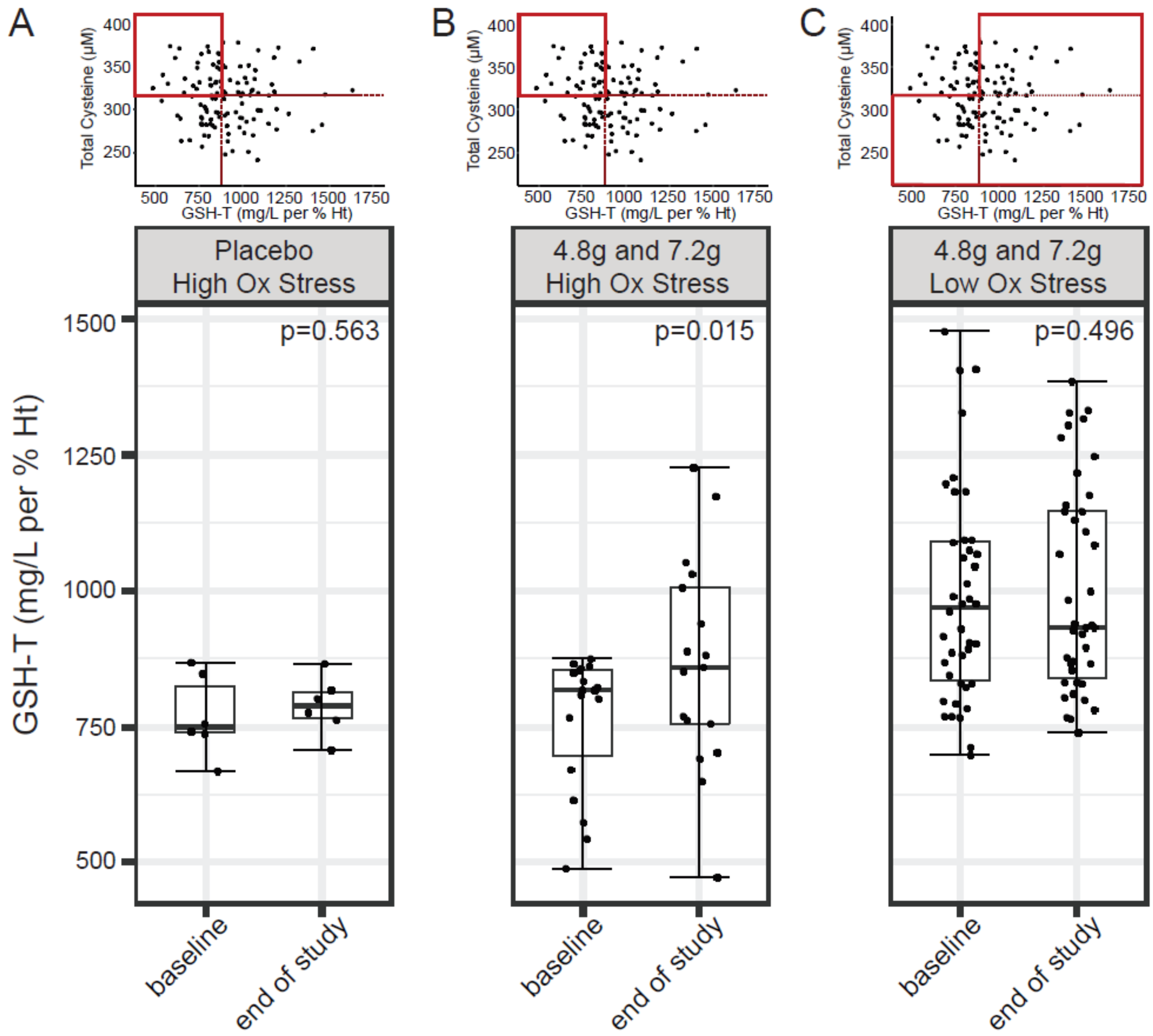

Supplementary Figure 3 Effect of GlyNAC supplementation on total glutathione levels in a subset of subjects with high basal levels of total cysteine and low basal levels of total glutathione

Post-hoc subset analysis of response to **(A)** placebo or **(B)** high doses of GlyNAC treatment (4.8g and 7.2g) in subjects characterized by elevated levels of oxidative stress (T-cysteine above the median) and low baseline glutathione values (GSH-T below the median) in fasted state at baseline and end of study. **(C)** Post-hoc subset analysis of response to high doses of GlyNAC treatment (4.8g and 7.2g) in subjects characterized by low oxidative stress in fasted state at baseline and end of study. Boxplots show the median, the first to third quartile, the 1.5x interquartile ranges, and outliers. p= p-value, nonparametric paired Wilcoxon/Mann-Whitney tests of medians.

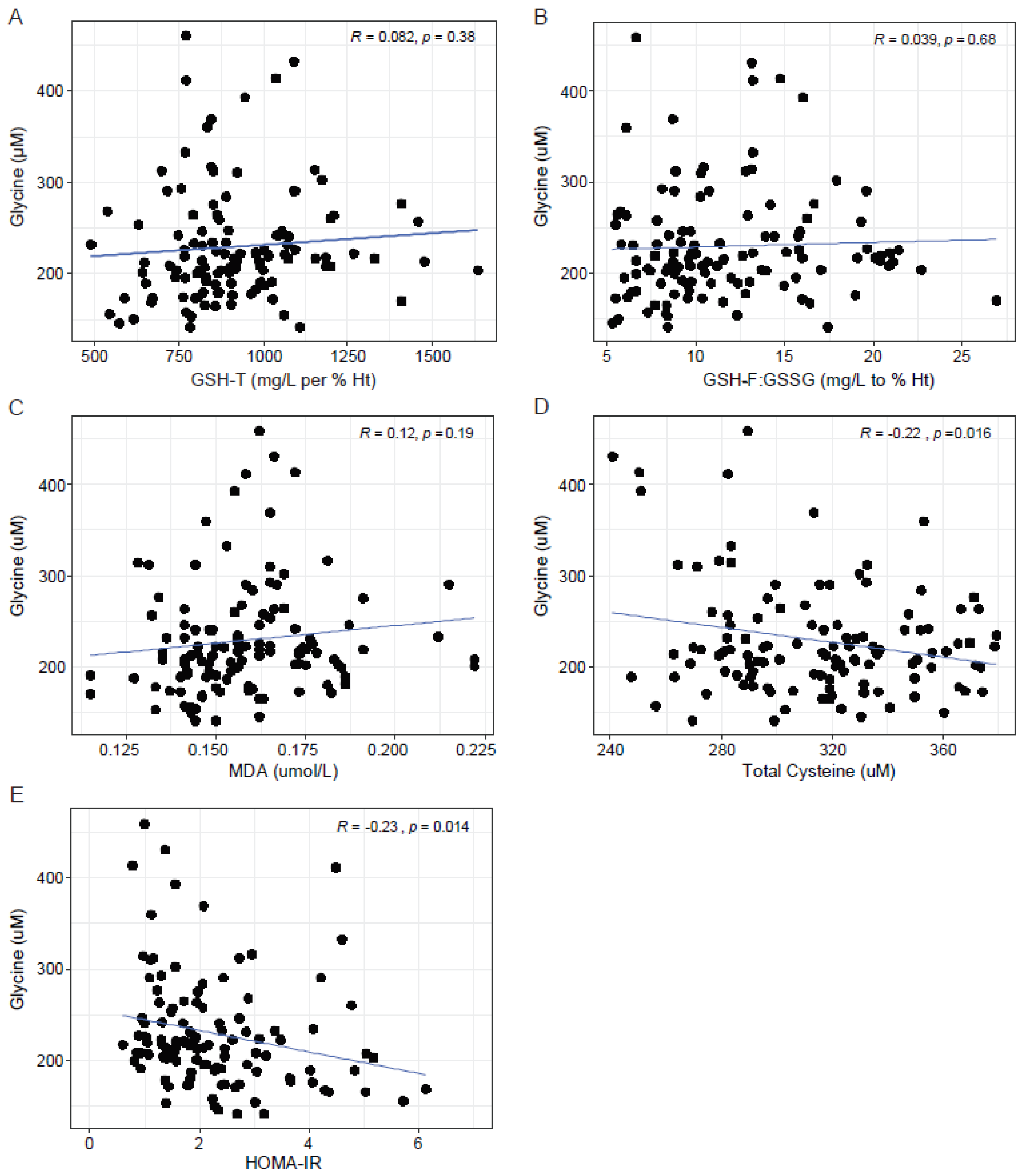

Supplementary Figure 4 Correlation of basal parameters of oxidative stress and metabolic impairment with plasma glycine levels in older adults

Correlation between basal levels of glycine measured in plasma and **(A)** GSH-T, **(B)** GSH-F:GSSG, **(C)** MDA, **(D)** total cysteine and **(E)** HOMA-IR in healthy older adults. R= Pearson's linear coefficient, p= p-value.

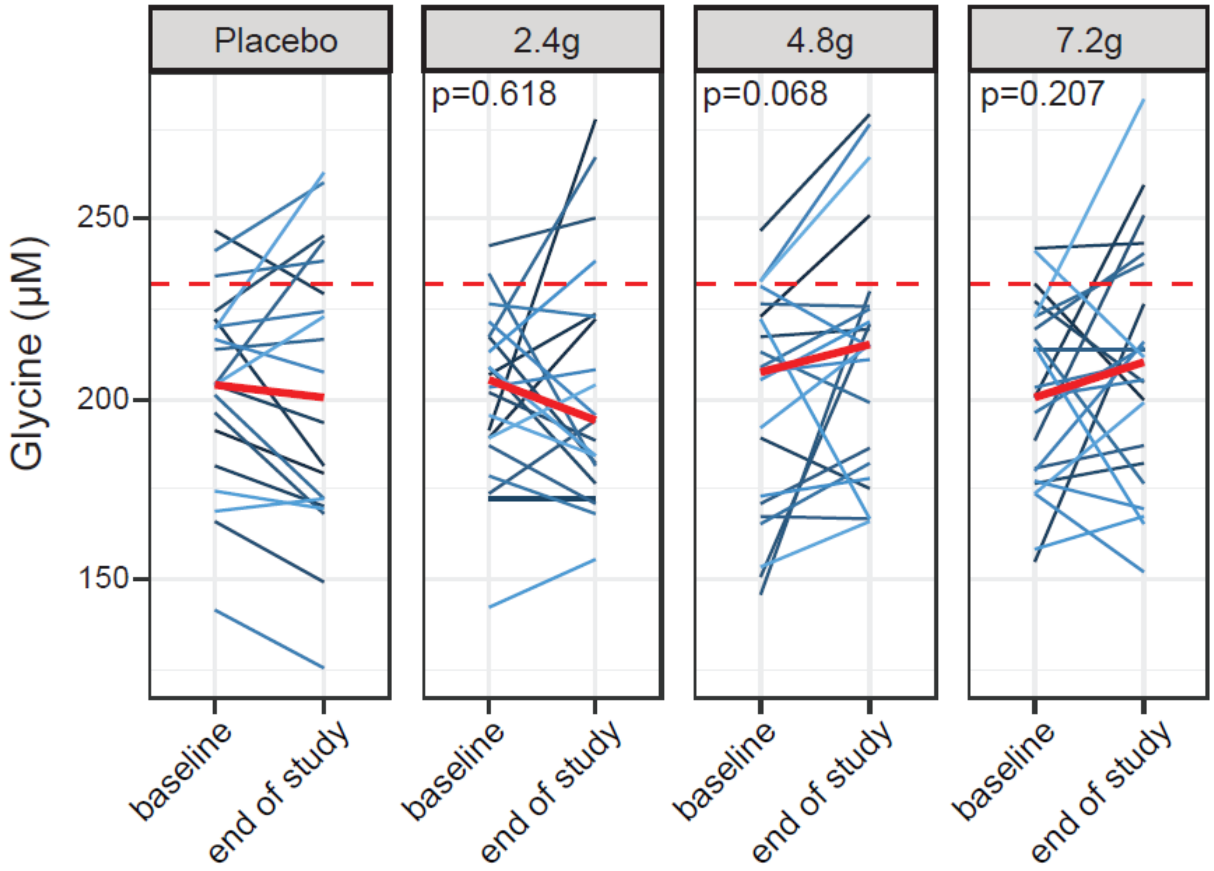

Supplementary Figure 5 Dose dependent effects of GlyNAC supplementation on glycine status

Effects of daily intake of placebo or different doses of GlyNAC on plasma glycine levels in a subpopulation of older adults (excluding the group above the third quartile). Glycine levels have been compared before (baseline, Visit 2) and after the 2-weeks of treatment (end of study, Visit 4) in samples taken prior to the dosing with GlyNAC (-60min). The red dashed lines represent the median of the young adults. p= p-value, linear mixed model testing intra-individual changes from baseline and end of study within each dose group and from placebo group.

2 Supplementary Tables

Supplementary Table 1 Dose composition

Composition of each sachet and dosing intervals.

**Daily Dose**	**Sachets**		Active (g)			Placebo (g)
			**Arm A**	**Arm B**	**Arm C**	Arm D
		Ingredients				
**Morning dose**	Sachet 1	N-acetylcysteine	0.6 g	0.6 g	0.6 g	**-**
		Glycine	0.6 g	0.6 g	0.6 g	**-**
		Isomaltulose	-	-	-	**1.2 g**
	Sachet 2	N-acetylcysteine	0.6 g	0.6 g	-	**-**
		Glycine	0.6 g	0.6 g	-	**-**
		Isomaltulose	-	-	1.2 g	**1.2 g**
	Sachet 3	N-acetylcysteine	0.6 g	-	-	**-**
		Glycine	0.6 g	-	-	**-**
		Isomaltulose	-	1.2 g	1.2 g	**1.2 g**
**Evening dose**	Sachet 1	N-acetylcysteine	0.6 g	0.6 g	0.6 g	**-**
		Glycine	0.6 g	0.6 g	0.6 g	**-**
		Isomaltulose	-	-	-	**1.2 g**
	Sachet 2	N-acetylcysteine	0.6 g	0.6 g	-	**-**
		Glycine	0.6 g	0.6 g	-	**-**
		Isomaltulose	-	-	1.2 g	**1.2 g**
	Sachet 3	N-acetylcysteine	0.6 g	-	-	**-**
		Glycine	0.6 g	-	-	**-**
		Isomaltulose	**-**	**1.2 g**	**1.2 g**	**1.2 g**

Supplementary Table 2 Exclusion Criteria

**Subject exclusion criteria**	**Non-interventional cohort**	1. Receipt of any medicinal product or nutritional product in clinical development within 30 days before enrolment in this trial.<br>2. Any history or presence of clinically relevant comorbidity, as judged by the Investigator.<br>3. Signs of acute illness as judged by the Investigator.<br>4. Any serious systemic infectious disease during four weeks prior enrolment in this trial<br>5. Clinically significant abnormal screening laboratory tests, as judged by the Investigator.<br>6. AST and/or ALT > 2 times the upper limit of normal.<br>7. Elevated serum creatinine values above the upper limit of normal.<br>8. Systolic blood pressure < 90 mmHg or >139 mmHg and/or diastolic blood pressure < 50 mmHg or >89 mmHg (excluding white-coat hypertension; therefore, a repeat test showing results within range will be acceptable).<br>9. Heart rate at rest outside the range of 50-90 beats per minute.<br>10. Clinically significant abnormal standard 12-lead electrocardiogram (ECG) after 5 minutes resting in supine position at screening, as judged by the Investigator.<br>11. Significant history of alcoholism or drug abuse as judged by the Investigator consuming more than 24 grams alcohol/day (for males), 12 grams alcohol/day (for females) on average.<br>12. Smoking or use of nicotine substitute products.<br>13. Any medication (prescription and non-prescription drugs) within 14 days before screening.<br>14. Blood donation or blood loss of more than 500 mL within the last 3 months prior to screening.<br>15. Mental incapacity, unwillingness or language barriers precluding adequate understanding or co-operation.<br>16. If female, pregnant or breast-feeding.<br>17. Consumption of high protein supplements within 60 days of screening and during the study.<br>**18.** Consumption of any antioxidant, vitamins, and herbals (see chapter 12.2) supplements within 2 weeks prior to screening and during the study.

	**Interventional cohort**	1. Known or suspected hypersensitivity to any component of the trial products.<br><br>2. Receipt of any medicinal product or nutritional product in clinical development within 30 days before randomisation in this trial.<br><br>3. History of multiple and/or severe allergies to drugs or foods or a history of severe anaphylactic reaction.<br><br>4. Any history or presence of clinically relevant comorbidity, as judged by the investigator.<br><br>5. Signs of acute illness as judged by the Investigator.<br><br>6. Any serious systemic infectious disease during four weeks prior to first intake of the trial product, as judged by the Investigator.<br><br>7. Clinically significant abnormal screening laboratory tests, as judged by the Investigator.<br><br>8. AST and/or ALT > 2 times the upper limit of normal.<br><br>9. Elevated serum creatinine values above the upper limit of normal.<br><br>10. Systolic blood pressure < 90 mmHg or >139 mmHg and/or diastolic blood pressure < 50 mmHg or >89 mmHg (excluding white-coat hypertension; therefore, a repeat test showing results within range will be acceptable).<br><br>11. Heart rate at rest outside the range of 50-90 beats per minute.<br><br>12. Clinically significant abnormal standard 12-lead electrocardiogram (ECG) after 5 minutes resting in supine position at screening, as judged by the Investigator.<br><br>13. Significant history of alcoholism or drug abuse as judged by the Investigator consuming more than 24 grams alcohol/day (for males), 12 grams alcohol/day (for females) on average.<br><br>14. Smoking more than 5 cigarettes or the equivalent per day.<br><br>15. Inability or unwillingness to refrain from smoking and use of nicotine substitute products 3 days prior and during the intervention.<br><br>16. Tested positive for Hepatitis Bs antigen.<br><br>17. Tested positive for hepatitis C antibodies.<br><br>18. Positive result to the test for HIV-1/2 antibodies or HIV-1 antigen.<br><br>19. Any medication (prescription and non-prescription drugs) within 14 days before test product intake with the exception of stable therapy with thyroid hormones, anti-hypertensive medication (except beta blockers) and if

		female with the exception of hormonal contraception or menopausal hormone replacement therapy.<br><br>20. Blood donation or blood loss of more than 500 mL within the last 3 months prior to screening<br><br>21. Mental incapacity, unwillingness or language barriers precluding adequate understanding or co-operation.<br><br>22. Consumption of high protein supplements within 60 days of screening and during the study.<br><br>23. Consumption of any antioxidants, vitamins and herbals (see chapter 12.2) supplement within 2 weeks prior to randomization and during the study.
**Day trial exclusion criteria**	**Visit 1 and 3**	1. Fasting (except intake of water) for less than 8 hours.<br><br>2. Strenuous exercise within the last 24 hours as judged by the investigator.<br><br>3. Any medical condition or AE that could interfere with the procedures of the study, as judged by the Investigator.
	**Visit 2 and 4 (with calorimetry)**	1. Fasting for less than 10 hours (up to 200 mL of water are allowed)<br><br>2. Strenuous exercise within the last 48 hours as judged by the investigator<br><br>3. Protein-rich meal in the evening before as judged by the investigator<br><br>4. Consumption of alcohol, caffeine- and/or methylxanthine-containing products in the last 24 hours before the measurement (i.e., coffee, coke, black/green tea, chocolate, cacao, energy drinks)

Supplementary Table 3 Safety outcomes

Safety panel parameters assessed before and after the treatment. A full list of safety outcomes is available as an additional data table within the supplementary information.

	Baseline	Baseline			End of study			
Dose	***Screening***	***Pre-dose***	***Post-dose (120)***	***P^a***	***Pre-dose***	***Post-dose (120)***	***P^c***	***P^e***
Systolic Blood Pressure (mmHg)								
**Placebo**	133.3 ± 7.25	134.1 ± 11.71		0.988	130.7 ± 7.49		0.528	
**2.4g**	131.9 ± 6.31	128.3 ± 13.39		0.641	126.8 ± 13.67		0.952	
**4.8g**	131.5 ± 7.10	131.3 ± 8.84		0.100	126.9 ± 10.24		0.278	
**7.2g**	131.7 ± 7.66	130.3 ± 11.05		0.960	130.6 ± 11.69		0.999	
Diastolic Blood Pressure (mmHg)								
**Placebo**	82.9 ± 5.52	80.2 ± 6.57		0.380	78.8 ± 6.24		0.838	
**2.4g**	83.1 ± 6.17	79.4 ± 6.55		0.231	77.4 ± 9.24		0.702	
**4.8g**	83.2 ± 5.63	80.4 ± 6.43		0.297	77.6 ± 6.59		0.343	
**7.2g**	84.7 ± 4.28	83.5 ± 6.62		0.769	79 ± 8.10		0.258	

Creatinine (µmol/L)								
**Placebo**		66.19 (61.42, 71.34)	64.01 (59.39, 68.98)	-	66.19 (61.42, 71.34)	64.33 (59.69, 69.33)	0.998	-
**2.4 g**		64.08 (59.54, 68.98)	60.89 (56.57, 65.54)	-	64.94 (60.32, 69.91)	61.93 (57.52, 66.67)	0.453	0.545
**4.8 g**		71.30 (66.33, 76.65)	69.01 (64.20, 74.19)	-	71.87 (66.84, 77.28)	69.37 (64.52, 74.59)	0.645	0.161
**7.2 g**		65.52 (60.95, 70.44)	63.22 (58.81, 67.96)	-	65.09 (60.55, 69.98)	61.79 (57.47, 66.44)	0.700	0.848
Glucose (mmol/L)								
**Placebo**		5.617 (5.37,5.88)	6.143 (5.87,6.43)	-	5.532 (5.29,5.79)	5.99 (5.73,6.27)	0.504	-
**2.4g**		5.397 (5.16,5.64)	6.138 (5.87,6.42)	-	5.408 (5.17,5.66)	6.226 (5.92,6.51)	0.925	0.483
**4.8g**		5.718 (5.47,5.97)	6.450 (6.18,6.74)	-	5.674 (5.43,5.93)	6.305 (6.03,6.59)	0.730	0.429
**7.2g**		5.614 (5.37,5.86)	6.275 (6.01,6.55)	-	5.509 (5.27,5.75)	6.419 (6.14,6.71)	0.394	0.897
Insulin (mU/L)								
**Placebo**		8.18 (6.61,10.12)	34.95 (28.25,43.24)	-	7.74 (6.26,9.58)	34.79 (28.12,43.04)	0.523	-
**2.4g**		6.65 (5.4,8.2)	28.93 (23.47,35.66)	-	6.68 (5.41,8.24)	33 (26.73,40.74)	0.966	0.331
**4.8g**		9.07 (7.38,11.14)	40.59 (33.05,49.86)	-	9.76 (7.93,12)	37.83 (30.75,46.53)	0.390	0.127
**7.2g**		8.74 (7.12,10.74)	33.51 (27.28,41.16)	-	8.35 (6.8,10.25)	34.82 (28.31,42.84)	0.582	0.618

TGs (mmol/L)								
**Placebo**	1.351 (1.16,1.58)	1.581 (1.35,1.85)	-	1.214 (1.04,1.41)	1.422 (1.22,1.66)	0.029	-
**2.4g**	1.087 (0.93,1.27)	1.242 (1.07,1.45)	-	0.989 (0.85,1.15)	1.207 (1.04,1.41)	0.052	0.066
**4.8g**	1.227 (1.06,1.43)	1.4 (1.21,1.63)	-	1.204 (1.04,1.4)	1.436 (1.23,1.67)	0.698	0.940
**7.2g**	1.198 (1.03,1.39)	1.360 (1.17,1.58)	-	1.208 (1.04,1.4)	1.369 (1.17,1.59)	0.859	0.963
ALP (U/L)							
**Placebo**	61.60 (56.73, 66.89)	61.95 (57.05, 67.27)	-	60.67 (55.87, 65.88)	61.14 (56.3, 66.38)	0.262	-
**2.4g**	63.97 (58.99, 69.37)	64.01 (59.03, 69.41)	-	65.26 (60.18, 70.77)	65.06 (59.99, 70.55)	0.143	0.216
**4.8g**	68.70 (63.44, 74.39)	68.80 (63.54, 74.50)	-	70.66 (65.25, 76.52)	70.93 (65.49, 76.81)	0.035	0.009
**7.2g**	67.72 (62.54, 73.34)	67.60 (62.42, 73.19)	-	70.52 (65.12, 76.36)	70.13 (64.76, 75.95)	0.002	0.010
AST (U/L)							
**Placebo**	20.10 (18.33, 22.03)	20.07 (18.31, 22.01)	-	19.06 (17.38, 20.89)	18.69 (17.05, 20.49)	0.115	-
**2.4g**	18.75 (17.13, 20.52)	18.80 (17.18, 20.58)	-	18.96 (17.32, 20.77)	18.83 (17.19, 20.62)	0.737	0.942
**4.8g**	21.20 (19.40, 23.17)	21.72 (19.88, 23.74)	-	21.54 (19.7, 23.55)	21.22 (19.41, 23.20)	0.635	0.061
**7.2g**	20.49 (18.75, 22.39)	20.41 (18.68, 22.31)	-	20.90 (19.13, 22.84)	20.46 (18.71, 22.37)	0.539	0.156

Values are expressed as LS-Means with 95% Confidence Interval. Statistics have been performed using a linear mixed model. *P^a=padj-value pre-dose vs screening, P^c= p-value for change from baseline to end of study at pre-dose, P^e= p-value end of study at pre-dose comparing placebo vs active dose group*

Supplementary Table 4 Effects of GlyNAC supplementation on oxidative stress markers

Acute and chronic changes in MDA and total cysteine (Cysteine-T) in whole blood

	Baseline			End of study				
Dose	*Pre-dose*	*Post-dose*	P^a	*Pre-dose*	*Post-dose*	P^a	P^c	P^e
MDA (µmol/L)								
**Placebo**	0.163 (0.155,0.172)			0.160 (0.152,0.168)			0.477	-
**2.4g**	0.154 (0.146,0.161)			0.152 (0.145,0.160)			0.727	0.156
**4.8g**	0.156 (0.148,0.164)			0.160 (0.153,0.168)			0.302	0.975
**7.2g**	0.156 (0.149,0.164)			0.154 (0.146,0.161)			0.536	0.249
Cysteine-T (µmol/L)								
**Placebo**	401.1 (374.4,429.8)	381.7 (356.3,408.9)	0.013	405.7 (378.7,420.4)	395.8 (369.4,424)	0.178	0.613	-
**2.4g**	421.4 (393.8,450.9)	400.2 (374,428.2)	0.006	417.8 (390.4,447.2)	402.3 (375.9,430.6)	0.053	0.701	0.549
**4.8g**	389.5 (364.4,416.3)	384.0 (359.3,410.5)	0.757	382.7 (357.9,405.7)	382.8 (358,409.2)	0.690	0.415	0.233
**7.2g**	415.9 (389.1,444.6)	414.7 (388.0,443.2)	0.923	396.7 (371.1,424)	410.6 (384,438.9)	0.039	0.028	0.646

Values for MDA and Cys-T were normalized to hematocrit and expressed as LS-Means in µmol/L with 95% Confidence Interval. MDA, malondialdehyde; Cysteine-T, total cysteine disulfides; *P^a=p-value pre-dose vs post-dose, P^c= p-value for change from baseline to end of study at pre-dose, P^e= p-value end of study at pre-dose comparing placebo vs active dose group.*

Supplementary Table 5 Glycine changes after supplementation in a subpopulation of older adults in whole blood

	Baseline (Median [IQR])	*End of study (Median [IQR])*	*Increment to placebo (%)*	*Increment to baseline*
**Placebo**	704.13 [166.74]	681.08 [134.26]	-	-
**2.4 g**	718.92 [146.33]	729.66 [227.50]	7.13	1.49
**4.8g**	660.90 [186.41]	738.66 [132.99]	8.45	11.77
**7.2g**	684.43 [117.5]	770.43 [137.36]	13.12	12.57

Supplementary Table 6 Statistical analysis results of the effects of glycine supplementation on glycine in whole blood

	Estimate	*Std error*	*t- value*	*Pr(>|t|)*	
**Intercept**	52.88	70.66	0.748	0.456	
**Baseline Glycine**	0.919	0.093	9.855	1.63e-15	***
**2.4 g**	29.99	27.03	1.110	0.27	
**4.8g**	65.10	27.63	2.356	0.021	*
**7.2g**	74.90	27.29	2.745	0.007	**

*Sign. codes: 0 '***' 0.001 '**' 0.01 '*' 0.05 '.' 0.1 ' ' 1*
Residual standard error: 86.98 on 81 degrees of freedom
Multiple R-squared: 0.559, Adjusted R-squared: 0.537
F-statistic: 25.68 on 4 and 81 DF, p-value: 9.307e-14

3 ANNEX

Safety panel parameters

Safety Panel 1

Hematology

Hematocrit	Leucocytes
Hemoglobin	Neutrophile granulocytes (total count and relative)
Erythrocytes	Lymphocytes (total count and relative)
Mean corpuscular volume (MCV)	Monocytes (total count and relative)
Mean corpuscular hemoglobin (MCH)	Eosinophile granulocytes (total count and relative)
Mean corpuscular hemoglobin concentration (MCHC)	Basophile granulocytes (total count and relative)
Thrombocytes (platelets)	

Biochemistry

Sodium	Uric acid
Potassium	Total protein
Calcium	Albumin
Chloride	Total bilirubin
Phosphate	Creatine kinase
Creatinine	Alkaline phosphatase
Urea	Gamma-glutamyltransferase (γ-GT)
AST (aspartate aminotransferase, GOT)	Lactic dehydrogenase (LDH)
ALT (alanine aminotransferase, GPT)	C-reactive protein
Total cholesterol	High-density lipoprotein (HDL) cholesterol
Low-density lipoprotein (LDL) cholesterol	Triglycerides

Coagulation (screening only)

International normalized ratio (INR)	Activated partial thromboplastin time (APTT)

Infectious serology (screening only)

Hepatitis B surface antigen	HIV-1/2 combi

Hepatitis C antibodies	
**Other**	
HbA1c (screening only)	β-HCG (females only; screening of young control group only)

Safety Panel 2

**Hematology**	
Hematocrit	Mean corpuscular hemoglobin concentration (MCHC)
Hemoglobin	Thrombocytes (platelets)
Erythrocytes	Leucocytes
Mean corpuscular volume (MCV)	
Mean corpuscular hemoglobin (MCH)	
**Biochemistry**	
Sodium	AST (aspartate aminotransferase, GOT)
Potassium	Alkaline phosphatase
Creatinine	Gamma-glutamyltransferase (γ-GT)
Glucose	Insulin
Total cholesterol	Triglycerides

Note: laboratory tests included in safety panel 2 are to be performed in conjunction with mixed meal for safety and compliance check (check of fasting state).
